# Imaging Trans-Cellular Neurexin-Neuroligin Interactions by Enzymatic Probe Ligation

**DOI:** 10.1371/journal.pone.0052823

**Published:** 2013-02-14

**Authors:** Daniel S. Liu, Ken H. Loh, Stephanie S. Lam, Katharine A. White, Alice Y. Ting

**Affiliations:** Department of Chemistry, Massachusetts Institute of Technology, Cambridge, Massachusetts, United States of America; Stanford, United States of America

## Abstract

Neurexin and neuroligin are transmembrane adhesion proteins that play an important role in organizing the neuronal synaptic cleft. Our lab previously reported a method for imaging the trans-synaptic binding of neurexin and neuroligin called BLINC (Biotin Labeling of INtercellular Contacts). In BLINC, biotin ligase (BirA) is fused to one protein while its 15-amino acid acceptor peptide substrate (AP) is fused to the binding partner. When the two fusion proteins interact across cellular junctions, BirA catalyzes the site-specific biotinylation of AP, which can be read out by staining with streptavidin-fluorophore conjugates. Here, we report that BLINC in neurons cannot be reproduced using the reporter constructs and labeling protocol previously described. We uncover the technical reasons for the lack of reproducibilty and then re-design the BLINC reporters and labeling protocol to achieve neurexin-neuroligin BLINC imaging in neuron cultures. In addition, we introduce a new method, based on lipoic acid ligase instead of biotin ligase, to image trans-cellular neurexin-neuroligin interactions in human embryonic kidney cells and in neuron cultures. This method, called ID-PRIME for Interaction-Dependent PRobe Incorporation Mediated by Enzymes, is more robust than BLINC due to higher surface expression of lipoic acid ligase fusion constructs, gives stronger and more localized labeling, and is more versatile than BLINC in terms of signal readout. ID-PRIME expands the toolkit of methods available to study trans-cellular protein-protein interactions in living systems.

## Introduction

Neurexins (NRX) are presynaptic adhesion proteins that bind across the synaptic cleft to postsynaptic neuroligins (NLG). This trans-cellular binding is believed to play a role in synapse formation, specification, and/or stabilization [1]. To facilitate the study of NRX-NLG biology, it would be desirable to have a non-invasive method that reports on their binding in living cells. A recent study [2], building upon the GRASP technology (GFP Reconstitution Across Synaptic Partners) introduced earlier [3], identifies synapses using GFP complementation of the NRX-NLG interaction. A fragment of GFP is fused to the ectodomain of NRX while the complementary GFP fragment is fused to the ectodomain of NLG. Formation of a NRX-NLG adhesion complex at synapses recombines the GFP fragments, and fluorescence is restored an hour or more later. The primary limitations of GRASP for NRX-NLG interaction detection are that GFP recombination is irreversible [4] and GFP fluorescence is dim. The irreversibility can shift the equilibrium between the complexed and non-complexed states of NRX-NLG, and preclude dynamic reporting of NLG-NRX interactions upon stimulation.

**Figure 1:**
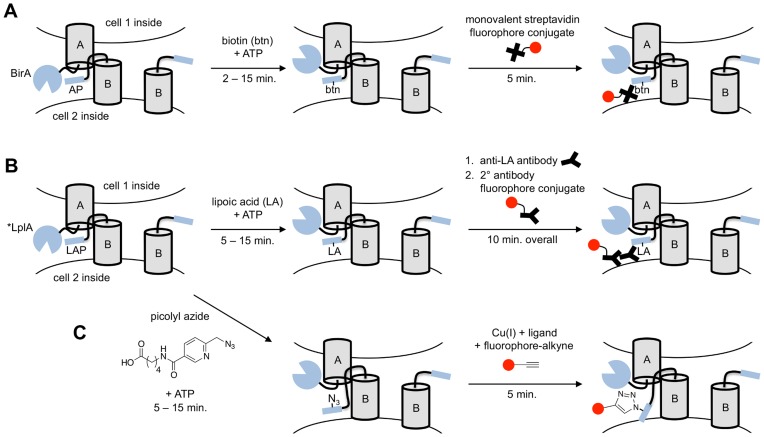
Scheme showing BLINC and ID-PRIME methods for imaging trans-cellular protein-protein interactions. (**A**) In Biotin Labeling of INtercellular Contacts (BLINC), protein A is genetically tagged with the 35 kDa *E. coli* biotin ligase (BirA) on the extracellular side. Protein B is genetically tagged with a 15-amino acid acceptor peptide (AP) for BirA. When proteins A and B interact, BirA ligates biotin onto protein B, which can be detected using a monovalent streptavidin-fluorophore conjugate [23]. (**B**) In Interaction-Dependent PRobe Incorporation Mediated by Enzymes (ID-PRIME), protein A is genetically tagged with a 38 kDa mutant of *E. coli* lipoic acid ligase (*LplA = W37A, T57I, F147L, H267R mutant of LplA) on its extracellular side. Protein B is genetically tagged with a 13-amino acid ligase acceptor peptide (LAP) for LplA. When proteins A and B interact, *LplA ligates lipoic acid onto protein B, which can be detected using an antibody-fluorophore conjugate. **(C)** Alternative ID-PRIME detection using picolyl azide ligation onto protein B. Ligated azide can be detected by copper-catalyzed click chemistry with alkyne-fluorophore conjugates [7].

In 2010, we presented an alternative approach to image trans-synaptic NRX-NLG interactions based on enzymatic biotinylation of an acceptor peptide (AP) by *E. coli* biotin ligase (BirA, Figure 1A) [5] (paper now retracted). In this report, AP was fused to NLG and BirA was fused to NRX. When expressed in different but contacting neurons, site-specific biotinylation, detected by staining of live neurons with streptavidin-fluorophore conjugates, was reported at synaptic contacts. This method was named BLINC, for Biotin Labeling of INtercellular Contacts [5]. Since this publication, we have discovered that the work in this paper cannot be reproduced. Here, we examine the technical reasons for irreproducibility, make changes in the BLINC constructs and protocols in order to achieve successful BLINC labeling in neuronal cultures, and then introduce an improved method for NRX-NLG contact imaging based on lipoic acid ligase instead of biotin ligase (Figures 1B–C).

## Materials and Methods

### Plasmids Summary

All genetic constructs used in this work are summarized in Table S1. For each construct, the domain organization, BirA, AP, LplA, or LAP insertion site, epitope tag, promoter, and vector are given. The table also indicates which plasmids were used in each figure. Constructs were prepared by standard restriction cloning methods and QuikChange mutagenesis (Stratagene). Overlap extension PCR was used to clone pCAG- BirA_36_-NRX3β and pCAG-BirA_272_-NRX3β.

### HEK Cell Culture and Transfection

Human embryonic kidney 293T (HEK) cells were grown in minimal essential medium (MEM, Mediatech) supplemented with 10% (v/v) fetal bovine serum (PAA Laboratories) at 37°C under 5% CO_2_. Cells were typically transfected at ∼70% confluence using Lipofectamine 2000 (Life Technologies) using the manufacturer’s suggested protocol. Cells for imaging were grown on 150 µm glass coverslips pre-coated with 50 µg/ml human fibronectin (Millipore). Approximately 24 hours after transfection, cells were lifted by trypsinization, co-plated at ∼80% density, and labeled for BLINC or ID-PRIME ∼24 hours later.

### Rat Hippocampal Neuron Culture and Lipofection

Sprague Dawley rat embryos were sacrificed at embryonic day 18. Dissected hippocampal tissue was digested with papain (Worthington) and DNaseI (Roche), then plated on 0.09–0.12 mm thickness glass coverslips (Carolina Biological Supply) in a 1∶1 volume ratio of growth medium A and growth medium B and cultured at 37°C under 5% CO_2_. Growth medium A is MEM (Sigma) with L-glutamine (Sigma) supplemented with 10% (v/v) fetal bovine serum (PAA laboratories) and 2% (v/v) B27 (Life Technologies). Growth medium B is Neurobasal medium (Life Technologies) supplemented with 2% (v/v) B27 and 1% (v/v) GlutaMAX (Life Technologies). Glass coverslips were pretreated with poly-D-lysine (Sigma) and mouse laminin (Life Technologies). At 2 days *in vitro*, half of the spent culture medium was replaced with fresh growth medium B. This process was then repeated every 24 hours starting at 5 days *in vitro*. Transfection by Lipofectamine 2000 was performed between 5 to 11 days *in vitro*, using 1 µL Lipofectamine 2000 reagent per 1.91 cm^2^ well (less than the manufacturer’s recommendation, to reduce toxicity).

### Ethics Statement

All animals were housed, cared for, and experiments conducted in accordance with the Massachusetts Institute of Technology Committee on Animal Care guidelines (Assurance # A-3125-01) as specifically approved as part of animal protocol # 0910-076-13. Pregnant Sprague Dawley rats were euthanized at embryonic day 18/19 using carbon dioxide asphyxiation. Euthanasia was considered complete when animals were unresponsive to tail pinch, according to the recommendations of the Panel on Euthanasia of the American Veterinary Medical Association (AVMA). After euthanasia, the thoracic cavity was opened or cervical dislocation was carried out. The rat embryos were removed from the uterus and decapitated to remove the brain. No pain was expected under this protocol because of the carbon dioxide used and the under-developed sensory nervous systems of the pups.

### Neuron Nucleofection Using the Rat Neuron Nucleofector Kit (Lonza)

Dissociated neurons were suspended in 100 µL nucleofection solution provided by the kit at a density of 1–1.5×10^7^ cells/mL, and mixed with 1 µg of each plasmid construct, then transferred into a nucleofection cuvette and nucleofected with the O-003 program. Cells were rescued in growth medium A (pre-warmed to 37°C), then plated onto poly-D-lysine treated glass coverlips (12 mm diameter) at a density of 150,000 cells per 1.9 cm^2^. We note that the homemade nucleofection solution reported in [5] works for some mammalian cell lines, but causes neuron sickness in our hands.

### BLINC Labeling of HEK and Neuronal Cultures

BLINC labeling was typically carried out 24 hours after co-plating for HEK cell cultures, or 5–12 days after co-plating for neuron cultures. Cells were incubated in growth medium B containing 20 µM biotin (gift from Tanabe USA), 500 µM ATP, and 1.25 mM magnesium acetate for 5–15 min. at 37°C. Cells were then rinsed three times with Tyrode’s buffer (145 mM NaCl, 1.25 mM CaCl_2_, 3 mM KCl, 1.25 mM MgCl_2_, 0.5 mM NaH_2_PO_4_, 10 mM glucose, 10 mM HEPES, pH 7.4) and subsequently stained with wild-type streptavidin-Alexa Fluor 647 (AF647) or monovalent streptavidin-AF647 conjugate [6] in Tyrode’s buffer supplemented with 0.5% (w/v) vitamin-free casein (MP Biomedicals) for 5 min. at 37°C. Cells were rinsed three more times with Tyrode’s buffer before imaging.

**Figure 2:**
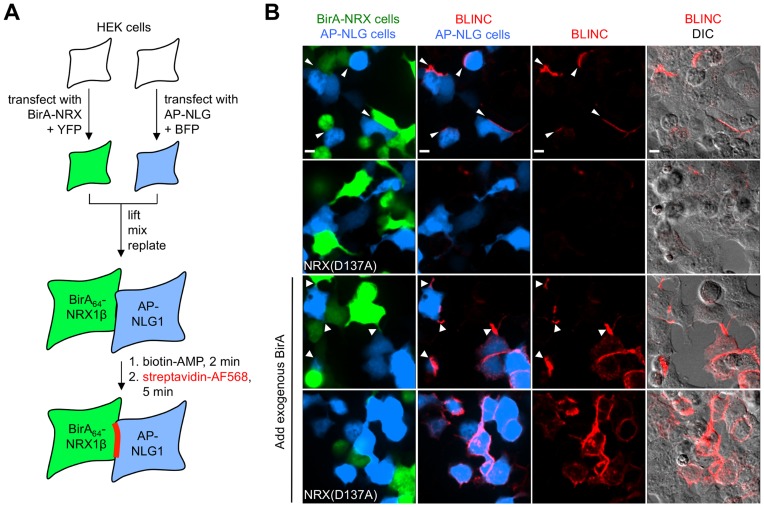
BLINC for imaging neurexin-neuroligin interactions in HEK cells. (**A**) Scheme showing the BLINC experimental protocol. Two pools of HEK cells were separately transfected with BirA_64_-NRX1β plus YFP, or AP-NLG1 plus BFP. The pools were then mixed and allowed to form contacts over 24 hours. BLINC labeling was performed with 10 µM biotin-AMP for 2 minutes (note that biotin+ATP was used instead for neuron cultures in other figures, for reasons explained in Figure S6). Biotinylated AP sites were detected by live-cell staining with streptavidin-AF568 for 5 minutes. (**B**) BLINC imaging results. Controls are shown with a D137A mutation in BirA-NRX to abolish its interaction with NLG (rows 2 and 4), and 1 µM exogenous BirA added during the biotin-AMP step to label total cell surface AP-NLG1 (rows 3 and 4). When a NLG-expressing cell apposes a NRX-expressing cell, BLINC signal is localized at contact sites (thin arrow heads, row 1). The same phenomenon was observed when exogenous BirA was added to label the total NLG pool (thick arrow heads, row 3). All scale bars, 10 µm.

Alternatively, HEK cells were treated with 5 µM biotin-AMP [6] for 2 min. at 37°C instead of biotin plus ATP (in Figure 2B). We found that labeling with biotin-AMP was suitable for HEK cells but not for neurons, as it produced high background (Figure S6).

Biotinylation of total surface AP using purified enzyme was performed in the same way except that 1 µM biotin ligase [6] was also added during the first labeling step.

### ID-PRIME Labeling of HEK and Neuronal Cultures

ID-PRIME labeling was typically carried out 24 hours after co-plating for HEK cell cultures, 5–12 days after co-plating for nucleofected neuron cultures, or 1–2 days after sequentially lipofecting neurons. Cells were treated with Tyrode’s buffer containing 500 µM ATP, 1.25 mM magnesium acetate, and either 100 µM DL-α-lipoic acid (Alexis Biochemicals) or 100 µM picolyl azide [7] for 15–20 min. at 37°C.

To detect lipoic acid, cells were rinsed three times in Tyrode’s buffer and subsequently stained with a 1∶200 dilution of rabbit anti-lipoic acid polyclonal antibody (Calbiochem) for 5 min. in the same buffer. Cells were again rinsed three times, followed by a 1∶300 dilution of goat anti-rabbit secondary antibody AF647 conjugate (Life Technologies) for the same time in the same buffer. Cells were imaged live after three further rinses.

To detect picolyl azide on HEK cells, cells were rinsed three times in Tyrode’s buffer and treated with 50 µM CuSO_4_, 2.5 mM sodium ascorbate, 20 µM alkyne-AF647 conjugate (Life Technologies), 250 µM THPTA ligand [8] and 100 µM 4-hydroxy-2,2,6,6-tetramethylpiperidin-1-oxyl (TEMPOL, Calbiochem) in the same buffer for 5 min. at room temperature. Ligand bound Cu(I) complexes were pre-formed by mixing the CuSO_4_, sodium ascorbate and THPTA, and incubating at room temperature for 10 min., before the alkyne and TEMPOL were added. Cells were rinsed three times further, then fixed with 4% (v/v) formaldehyde in a pH 7.0 buffer containing 0.12 M sucrose for 15 min. at room temperature before imaging.

Azide detection in neurons was performed after fixation (Figure S9). After picolyl azide ligation on living neurons as described above, neurons were fixed with 4% (v/v) formaldehyde in Tyrodes buffer, then blocked in Tyrode’s buffer supplemented with 0.5% (w/v) casein for one hour. Cells were treated with 1 mM CuSO_4_, 2.5 mM sodium ascorbate, 5 µM alkyne-AF647 conjugate, 100 µM TBTA ligand [8] and 100 µM TEMPOL in the same buffer for one hour at room temperature. Ligand bound Cu(I) complexes were pre-formed by mixing the CuSO_4_, sodium ascorbate and TBTA, and incubating at room temperature for 10 min., before the alkyne and TEMPOL were added. Cells were imaged after three further rinses.

### Immunofluorescence Detection of BirA and LplA Fusion Constructs in HEK and Neuronal Cultures

For live-cell immunofluorescence detection, cells were incubated with a 1∶200 dilution of either a mouse anti-c-Myc antibody (Calbiochem) or a rabbit anti-HA antibody (Rockland) in Tyrode’s buffer supplemented with 0.5% (w/v) casein for 15 min. at 37°C. Cell were rinsed three times with Tyrode’s buffer, and subsequently stained with the corresponding secondary antibody: goat anti-mouse-AF568 conjugate or goat anti-rabbit-AF568 conjugate (Life Technologies) in the same buffer. Cells were rinsed three times with Tyrode’s buffer before imaging live at room temperature.

For immunofluorescence detection on fixed cells, samples were fixed with 4% formaldehyde in Tyrode’s buffer, then permeabilized with methanol at −20°C. Cells were blocked for one hour in Tyrode’s buffer supplemented with 0.5% (w/v) casein, followed by primary antibody detection for one hour in the same buffer. A 1∶200 dilution of one of the following antibodies was used: mouse anti-c-Myc antibody (Calbiochem), rabbit anti-HA antibody (Rockland), or mouse anti-FLAG M2 antibody (Agilent). Cells were then rinsed three times with Tyrode’s buffer and subsequently stained with the corresponding secondary antibody: goat anti-mouse-AF488 conjugate, goat anti-rabbit-AF488 conjugate, or goat anti-rabbit-AF568 conjugate (Life Technologies). Cells were rinsed three times further with Tyrode’s buffer before imaging.

### Confocal Fluorescence Microscopy

Neuron cultures placed in Tyrode’s buffer or HEK cells placed in Dulbecco’s phosphate buffered saline (Gibco) were imaged using a Zeiss AxioObserver.Z1 inverted confocal microscope with a 40X oil-immersion objective. The microscope was equipped with a Yokogawa spinning disk confocal head, a Quadband notch dichroic mirror (405/488/568/647 nm), and 405 (diode), 491 (DPSS), 561 (DPSS), and 640 nm (diode) lasers (all 50 mW). BFP (405 nm laser excitation, 445/40 emission filter), GFP/Venus/AF488 (491 nm laser excitation, 528/38 emission filter), dsRed/tdTomato/AF568 (561 nm laser excitation, 617/73 emission filter), AF647 (640 nm laser excitation, 700/75 emission filter), and DIC images were collected using a Cascade II:512 camera and processed using SlideBook software version 5.0 (Intelligent Imaging Innovations). Acquisition time ranged from 10–2000 milliseconds. Neuron images in Figures S4 and S5 were projection summations from 0.5 µm-step optical stacks spanning 3.5 µm total depth.

### Quantification of Lipoic Acid ID-PRIME Sensitivity in Neurons

Analysis was performed on 10 fields-of-view using the SlideBook software. For each field-of-view, one binary mask was created for each of the two fluorescent protein transfection markers, Venus and tdTomato. The two masks were then intersected to create an intersection mask, which ranged from 26 to 172 puncta or oblong segments (totaling 741 from 10 fields-of-view). Maximum lipoic acid ID-PRIME pixel intensity was tabulated for the 741 puncta or segments, and those with ID-PRIME signal-to-noise ratio greater than 3∶1 were tallied, giving 38%. If the signal-to-noise ratio requirement was relaxed to 2∶1, then 54% of Venus/tdTomato overlaps were positive for ID-PRIME signal. Noise was defined as the averaged ID-PRIME intensity on three non-transfected cells. Microscope instrument noise, defined as the ID-PRIME intensity on an area of the glass coverslip with no cell coverage, was subtracted from both signal and noise before the signal-to-noise ratio was calculated.

### Chemicals and Reagents

The synthesis and characterization of biotin-AMP is described in Methods S1. ID-PRIME reagents for picolyl azide labeling are described in reference [7]. All chemicals were purchased from Sigma-Aldrich unless otherwise specified.

Additional experimental methods can be found in Methods S1.

## Results

### BLINC in HEK cells

We started by applying the constructs from the 2010 work [5] in human embryonic kidney 293T (HEK) cells (Figure 2). Two pools of HEK cells were separately transfected with the BirA_64_-NRX1β fusion (numbering indicates the BirA insertion site – at amino acid 64 of the immature NRX1β protein in this case) and the AP-NLG1 fusion. The two HEK populations were then resuspended, plated together, and allowed to form contacts over 24 hours. Trans-cellular biotinylation was initiated with the addition of biotin-AMP ester [5] for 2 min. Sites of AP biotinylation were detected on living cells by staining with streptavidin-AF568 conjugate (Figure 2A). Images in Figure 2B show biotinylation sites (BLINC signal) localized to NRX-NLG contacts, as indicated by the YFP and BFP co-transfection markers. AP-NLG1-expressing cells not contacting BirA cells were not labeled.

To test if BLINC labeling was interaction-dependent, we introduced a point mutation (D137A) in NRX to abolish Ca^2+^ binding [9] and therefore eliminate trans-interaction with NLG1. Figure 2B shows that the mutant construct, BirA_64_-NRX1β (D137A), gave almost no detectable BLINC staining at contact sites with AP-NLG1-expressing cells. As a positive control, we used exogenous BirA (purified BirA enzyme added to the cell media) to biotinylate the total surface pool of AP-NLG1, regardless of its proximity to a BirA-NRX1β-expressing cell. The third row in Figure 2B shows streptavidin staining of all AP-NLG1 expressing cells, not only those in contact with BirA-NRX1β-expressing cells. Interestingly, for blue cells in contact with green cells, the streptavidin signal was still localized to cell-cell contact sites, suggesting that when NRX and NLG expression levels are matched, their binding affinity is strong enough to aggregate the total surface protein pools at these contact regions. A similar control with BirA-NRX1β (D137A)-expressing cells also showed labeling of all AP-NLG1-expressing cells by exogenous BirA (fourth row), but the streptavidin signal was not localized to contact sites between green and blue cells. We believe that AP-NLG1 distributes evenly around the perimeter of the transfected cell because the NRX1β (D137A) mutant is unable to trap AP-NLG1 at contact sites. From this experiment, we conclude that NRX-NLG BLINC is robust and reproducible in HEK cells.

Unfortunately, we found that this was not the case in neuron cultures. After many unsuccessful efforts to reproduce neuron BLINC using the previously described NRX and NLG fusion constructs and protocols [5], we decided to systematically examine the fundamental aspects of the system.

### Problems with the CMV-AP-NLG1 Construct from Reference [5]

The design of the BLINC reporter system is such that AP and BirA fusions must be introduced into separate but contacting cells. HEK cells can be separately transfected, then lifted and replated together, but neurons cannot be replated without damaging their delicate processes and synapses. Therefore, it is necessary to transfect them immediately after dissociation, while they are still in suspension, and then plate them together thereafter. We previously opted for nucleofection-type transfection [5], because it is compatible with suspended neurons, gives high transfection efficiencies (necessary in order to see a reasonable number of overlapping BirA- and AP-containing processes), and eliminates the possibility of plasmid overlap, where BirA and AP fusions express together in the same neuron.

We first examined the expression of BLINC constructs in neurons by introducing the AP-NLG1 construct alone, using nucleofection, into suspended hippocampal neurons at 0 days *in vitro* (DIV0). Neurons were then plated, and five days later (since previous experiments were all reported at DIV5 and DIV16 [5]), we checked for expression by performing exogenous biotinylation with purified BirA added to the culture medium. This assay is expected to give a much stronger signal than any BLINC experiment, because BirA is provided in great excess, and total AP rather than just synaptic AP will be biotinylated. Figure S1 shows that no biotinylation was detected. We also found that biotinylation was undetectable at DIV12. We were unable to check at DIV16, because in order to see overlapping transfected processes at DIV5, it was necessary to plate neurons at a high initial density. As a result, neurons were often dense and unhealthy at DIV16, making it difficult to distinguish specific biotinylation from non-specific binding of streptavidin conjugates to unhealthy cells. This published AP-NLG1 construct [5] used a CMV promoter, which, according to previous reports, may give inconsistent [10] and activity-dependent expression in transiently transfected neuron cultures [11]. We therefore created an identical construct driven by the CAG promoter [12] (CMV enhancer/chicken β-actin) instead. This promoter has been used previously for strong transgene expression in neurons [13], [14]. Using CAG-AP-NLG1, we were able to detect weak but specific biotinylation in some neurons at DIV5 and DIV12 (Figure S1).

In another effort to improve the signal, we used a construct with three tandem AP tags: CAG-3xAP-NLG1. Figure S1 shows that exogenous biotinylation of neurons nucleofected with this construct produced signal well above background and specific to transfected cells, at both DIV5 and DIV12. All these comparisons were performed in parallel under identical conditions. We conclude that the CAG rather than CMV promoter was essential to give long-lasting (>5 day) expression of this fusion construct in transiently transfected neuron cultures, and the 3xAP rather than 1xAP tag on NLG was necessary to give streptavidin signal above background in the majority of neurons at both DIV5 and DIV12. The previous CMV-AP-NLG1 [5] construct lacked both persistent expression and detectability above background, which explains why it cannot be successfully used for BLINC in neurons.

### Problems with the CMV-BirA-NRX1β Construct from Reference [5]

We next turned our attention to the BirA-NRX fusion construct. We introduced the previously published construct, CMV-BirA_64_-NRX1β [5], by nucleofection into DIV0 hippocampal neurons. Figure S2 shows that expression was not detected by anti-c-Myc staining at both DIV5 and DIV12, although positive controls with the same construct introduced one day before labeling, by lipofection instead, were detectable.

We reasoned again that the CMV promoter could be part of the problem, so we switched to a CAG promoter. Figure S3 shows that CAG-BirA-NRX could be detected 4 days after lipofection of neuron cultures whereas CMV-BirA-NRX could not. Based on these experiments, we conclude that the CAG promoter is essential to give persistent expression of this construct as well, and the previously published CMV-BirA_64_-NRX1β [5] was undetectable in neuron cultures five days after its introduction by nucleofection. This explains why it cannot be successfully used for BLINC in neurons.

### New BirA Fusion Constructs

Using the improved CAG-BirA-NRX1β and CAG-3xAP-NLG1 constructs that give persistent expression in neurons after nucleofection, we attempted BLINC in neurons again, but were still unsuccessful. Based on the images in Figure S3, we suspected that part of the problem might be the poor trafficking of the BirA-NRX fusion to the cell surface and to synapses; the majority of it appeared to be intracellular and localized to the cell body rather than distal processes. Indeed, immunofluorescence staining in HEK cells (Figure S4C) showed that BirA_64_-NRX1β was mostly trapped in the secretory pathway compared to c-Myc-LAP-NRX1β (tag size 35 kD vs. 2.6 kD), suggesting that the large BirA tag disrupted trafficking, and that its insertion site would have to be optimized. Previous studies have inserted large tags, such as fluorescent proteins, into the cytosolic tail of NRX [15], [16] or into its stalk domain [17], [18], an extracellular region proximal to the transmembrane segment.

We wondered if moving the BirA tag to different locations might improve the surface targeting of our NRX fusion. We prepared two new extracellular fusions of BirA to the NRX3β gene. NRX3β is in many ways functionally interchangeable with NRX1β; the two isoforms display similar endogenous localization in neurons, possess similar trans-cellular binding affinity to NLG1 [19], and their crystal structures can be overlaid without significant differences [19], [20]. Figure S4C shows immunofluorescence staining of BirA_36_-NRX3β (*N*-terminal fusion, after signal peptide) and BirA_272_-NRX3β (stalk domain fusion), compared to HA-NRX3β, in HEK. Again, the BirA fusions were impaired in their surface targeting compared to HA-NRX3β, where the latter was predominantly localized to the cell surface. We also prepared a BirA fusion to NLG1 at its *N*-terminus after the signal peptide (BirA_48_-NLG1) and found that it too was largely trapped inside the cell compared to HA-AP-NLG1 (tag size 35 kD vs 2.9 kD) (Figure S4C).

Nevertheless, we tested our three new BirA fusion constructs in neurons. Figure S4D shows live-cell immunostaining of DIV12 neurons transfected with each construct. HA-NRX3β produced a very strong signal specific to transfected neurons. BirA_36_-NRX3β and BirA_272_-NRX3β were much weaker, but still detectable above background. In contrast, surface expression of BirA_48_-NLG1 was undetectable. Figure S4E shows the same experiment but with immunofluorescence staining performed after neuron fixation to detect total protein pools. From these images it wa apparent that HA-NRX3β and AP-NLG1 proteins could be found in distal processes, while the BirA fusions were predominantly localized to the cell bodies. We concluded that while none of our fusion sites tolerate the 35 kD BirA tag well, the NRX fusions are better than the NLG fusion, so we proceeded to try BLINC experiments with these.

### BLINC in Neurons with New BirA and AP Fusion Constructs

**Figure 3:**
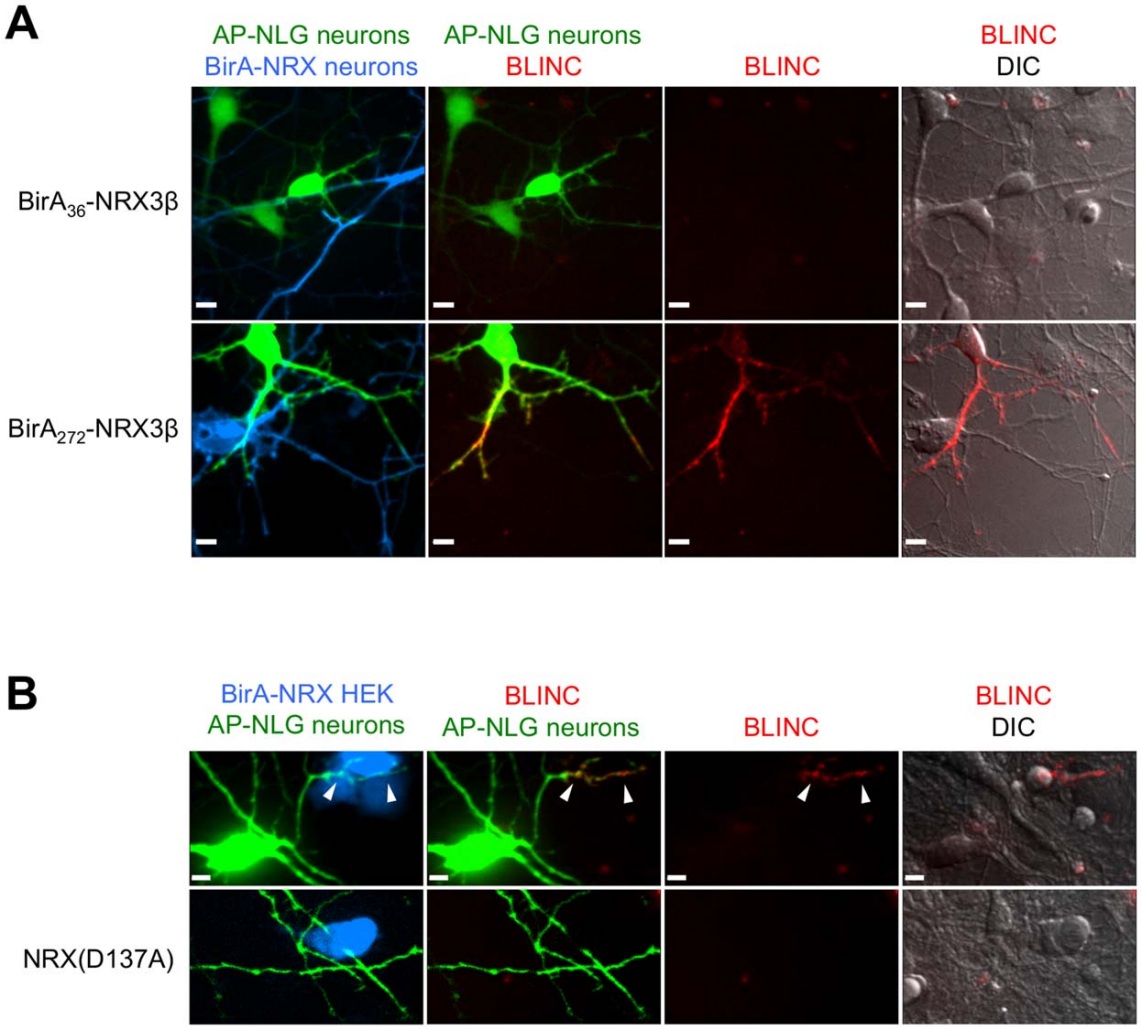
BLINC for imaging neurexin-neuroligin interactions in neuron cultures and in HEK-neuron mixed cultures. (**A**) BLINC labeling of pure neuron cultures. Two pools of hippocampal neurons were separately nucleofected at DIV0 with BirA-NRX plus a membrane tdTomato marker (shown in blue), or 3xAP-NLG1 plus a Venus marker (shown in green). For the top row, the BirA_36_-NRX3β construct was used, and for the bottom row the BirA_272_-NRX3β construct was used. All constructs had CAG promoters. Labeling was performed at DIV5 with biotin+ATP for 15 minutes, followed by monovalent streptavidin-AF647 detection for 5 minutes. Confocal images of live neurons showed no detectable BLINC signal for the BirA_36_-NRX3β fusion across 10 fields of view in which Venus- and Tomato-expressing neurons were observed to be crossing. For the BirA_272_-NRX3β fusion (bottom row), BLINC signal was detected in 5 out of 10 such fields of view. (**B**) BLINC labeling of mixed HEK-neuron cultures. HEK cells expressing BirA_272_-NRX3β and a dsRed marker (shown in blue) were plated on top of rat hippocampal neurons transfected with lipofectamine at DIV10 with 3xAP-NLG1 plus a Venus marker (shown in green). Labeling was performed at DIV11 as in (A). BLINC signal could be detected in 22 out of 30 fields of view, and was localized to contact sites (arrow heads). The bottom row shows a control with a D137A mutation in NRX3β; BLINC signal was not observed in any field of view. All scale bars, 10 µm.

First, we verified that both BirA_36_-NRX3β and BirA_272_-NRX3β gave detectable and localized trans-cellular BLINC labeling with 3xAP-NLG1 in HEK cultures (data not shown), demonstrating that they are functionally competent. Then we performed a BLINC experiment in hippocampal neurons, introducing each construct into separate pools of suspended DIV0 neurons by nucleofection. Figure 3A shows that whereas BirA_272_-NRX3β produced BLINC labeling at sites of overlap with 3xAP-NLG1-expressing neurons (indicated by overlap of green and blue transfection markers), BirA_36_-NRX3β did not. This trend was observed across >15 fields of view in this experiment. The observation that the site of BirA insertion into the extracellular domain of NRX3β influenced BLINC sensitivity is interesting in light of the fact that no difference in signal between these two constructs was seen in HEK cultures (data not shown). Perhaps the presence of endogenous NRX interaction partners in neurons alters NRX’s conformation and decreases the steric accessibility of fused BirA_36_, but not BirA_272_. The immunofluorescence controls in Figure S4D show that the difference in BLINC outcomes in neurons cannot be explained by a difference in surface expression levels for BirA_36_-NRX3β versus BirA_272_-NRX3β.

We noticed that the BLINC signal in neurons was strongest at sites of overlap between transfected cells, but streptavidin staining was also detected at non-overlapping regions on the 3xAP-NLG1-expressing cell (Figure 3A), suggesting that AP-NLG biotinylated in *trans* by BirA could subsequently diffuse away from the site of interaction. This contrasted with our observations in HEK cells, where the BLINC signal was tightly localized to cell-cell contacts and not diffusive (Figure 2B). We hypothesized that this discrepancy in localization of BLINC signal resulted from a difference in BirA:AP stoichiometry in these two experimental configurations. Since the insertion of BirA into NRX3β strongly impeded NRX3β trafficking to the surface of neurons while AP-NLG1 did not have an apparent trafficking defect (Figure S4D–E), there was probably insufficient BirA-NRX3β to anchor biotinylated 3xAP-NLG1, and the latter could diffuse away from the contact site after biotinylation. This explanation is supported by our observations in a mixed culture experiment, where HEK cells expressing BirA_272_-NRX3β were plated on top of hippocampal neurons expressing 3xAP-NLG1 (Figure 3B). Here, the resulting BLINC signal tightly localized to contact sites and did not diffuse outward. We believe this is because the quantity of BirA-NRX3β presented on the surface of the overlaid HEK cell was much higher than that presented on the surface of an overlaid neuron, and therefore anchoring of the biotinylated 3xAP-NLG1 pool could occur. Using this mixed culture assay, we also performed a negative control with the non-interacting D137A mutant of NRX and observed an absence of BLINC signal (Figure 3B).

Another feature of our BLINC signal in neurons (Figure 3A) is that it is clearly not synaptic. Apposing neurons differentially expressing the transgenic NRX and NLG fusions sometimes “zipped up” along one another’s processes, establishing large zones of contact that were clearly not synapses. This is likely an overexpression artifact. Interestingly, when neurons were transfected with either BLINC construct alone, the constructs displayed good overlap with pre- and post-synaptic markers (Figure S5), but in a *trans* experiment, the affinity of the overexpressed fusion constructs for one another greatly perturbed neuron morphology.

### Optimization of BLINC Labeling Reagent

Since BirA has a higher affinity for the biotin-AMP intermediate than ATP [21], biotin-AMP can be supplied at micromolar concentrations and still produce labeling signal comparable to millimolar concentrations of ATP (+20 µM biotin) [6]. Initially, as per the reported protocol [5], we attempted BLINC labeling in neurons using biotin-AMP in place of biotin+ATP (Figure S6). This option is less likely to activate endogenous purine receptors on neurons and cause toxicity [22]. We observed, however, that even 2.5 µM biotin-AMP resulted in much higher background fluorescence on neurons than 20 µM biotin +1 mM ATP (Figure S6). Higher biotin-AMP concentrations worsened the background without increasing the BLINC signal. This problem was not observed in BLINC experiments in HEK cells (Figure 2B), so we surmise that the high-energy biotin-AMP reagent was covalently reacting with nucleophiles on polylysine/laminin-coated coverslips. Our observations are inconsistent with the previous BLINC study [5], in which 10 µM biotin-AMP was used without detectable background. We note, however, that biotin-AMP can be used successfully for biotinylation of total surface AP-NLG1 pools on neurons, as in reference [23], because the signal is so much stronger than BLINC signal that it can be clearly detected above the biotin-AMP-related background.

### NRX-NLG BLINC in Neurons is not Robust

Even with our improvements to the BLINC reporter constructs and labeling protocol, we found that BLINC labeling in neurons was not robust. BLINC signal intensity varied from nucleofection to nucleofection and sometimes was not detectable at all. We believe this is a consequence of variations in BirA-NRX surface expression levels – sometimes it fell below the threshold necessary to produce detectable labeling signal. We conclude that BLINC in its current form can be a powerful and robust tool for imaging NRX-NLG interactions in HEK cultures (as in Figure 2B) and in HEK-neuron mixed cultures (as in Figure 3B), but the technology in its current form is too unreliable in pure neuron cultures. For this reason, we turned our attention to an alternative methodology for NRX-NLG contact imaging in neurons.

### ID-PRIME for NRX-NLG Interaction Imaging

Our lab has previously developed a suite of methods for targeting chemical probes to specific proteins in living cells using engineered mutants of *E. coli* lipoic acid ligase (LplA). These methods are collectively called “PRIME”, for PRobe Incorporation Mediated by Enzymes [7], [24]–[27]. PRIME works in a similar way to BirA-mediated biotinylation, with the ligase catalyzing covalent conjugation of a small molecule to a recognition peptide (the Ligase Acceptor Peptide, or LAP), but our engineering of the LplA active site has made it possible to conjugate a wide range of chemical structures besides lipoic acid, including fluorophores [24], photocrosslinkers [26], and functional group handles [27]. We have also found that the LplA/LAP pair can be used for detection of cytosolic protein-protein interactions by ID-PRIME (Interaction-Dependent PRIME), when the affinity of LAP for LplA is tuned such that probe ligation occurs only when the proteins to which LplA and LAP are fused interact [28]. We wondered if the LplA/LAP pair could be used for detection of intercellular protein-protein interactions in a manner analogous to BLINC, as shown in Figures 1B–C.

There were a few considerations before we could attempt such an experiment. First, we previously observed that LplA and its mutants have high activity in the mammalian cytosol, but the activity drops for unknown reasons when LplA is targeted to the secretory pathway or the cell surface [24]. Separate efforts in our lab have produced, using yeast display evolution, a quadruple mutant of LplA with higher activity in the secretory pathway and on the cell surface [29]. Second, we considered which LAP sequence to use: the regular, high affinity sequence used for most PRIME experiments with a K_M_ of 13 µM [30], or the lower affinity sequence used for intracellular ID-PRIME with a K_M_ >200 µM [28]. We opted for the high affinity sequence because we predicted that the lower effective protein concentrations in a trans-cellular experiment would render PRIME still interaction-dependent – just as we observed BLINC to be interaction-dependent with regular AP (K_M_ of 25 µM [31]) (Figure 2B) even though a modified, lower-affinity AP(-3) peptide was previously necessary for interaction-dependent biotinylation by BirA in the cytosol [32]. Third, we considered which of many PRIME probes to use for trans-interaction readout at the cell surface. We selected the natural substrate lipoic acid (Figure 1B) and picolyl azide (Figure 1C). The former, detectable by antibody-fluorophore conjugates, is advantageous for its superior ligation kinetics compared to unnatural substrates [24], [25], [27]. The latter is attractive because detection of the picolyl azide is performed entirely with small-molecule reagents (“click” chemistry with alkyne-fluorophore conjugates [7]), which have better steric access to crowded cellular junctions, do not induce crosslinking, and minimize perturbation to the subsequent trafficking and internalization of labeled proteins compared to detection by antibodies or streptavidin.

### ID-PRIME in HEK Cells

**Figure 4:**
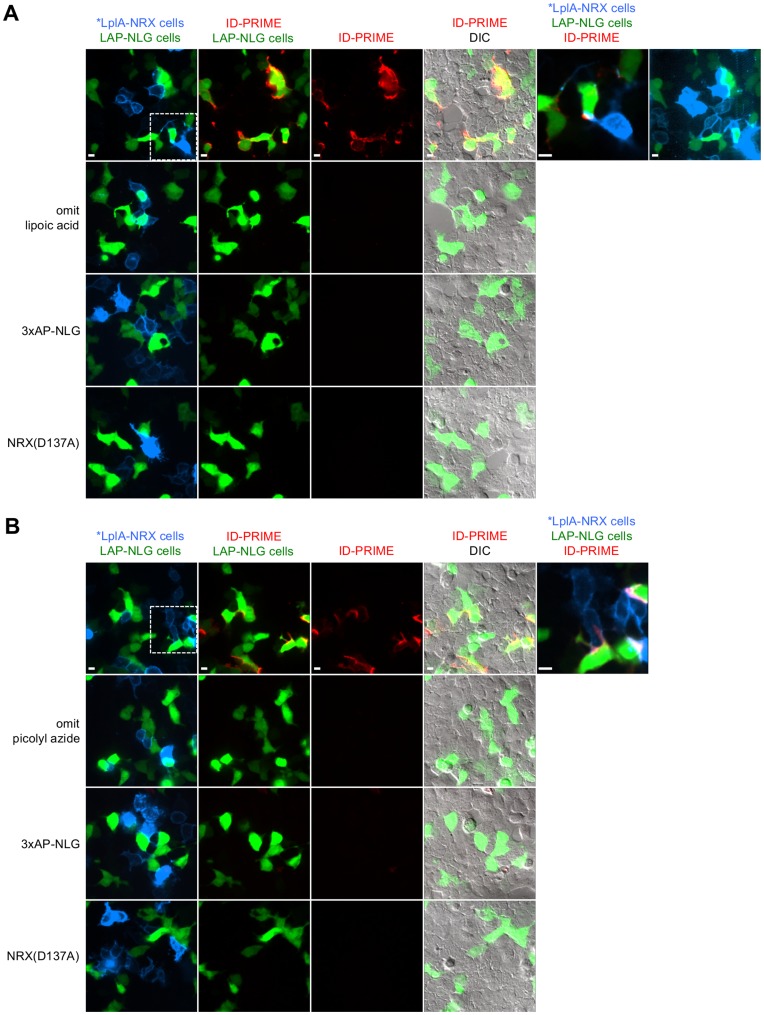
ID-PRIME for imaging neurexin-neuroligin interactions in HEK cells. (**A**) ID-PRIME with lipoic acid readout (as in Figure 1B). HEK cells were separately transfected with *LplA_36_-NRX3β plus a membrane-localized tdTomato marker (shown in blue), or 3xLAP-NLG1 plus a Venus marker. After mixing and replating, cells were labeled with 50 µM lipoic acid +500 µM ATP for 15 minutes. Ligated lipoic acid was detected with an anti-lipoic acid antibody followed by a secondary antibody-AF647 conjugate (shown in red) for 5 minutes each. For row 1, a magnified view representing the boxed region, and a more contrasted view of the transfection markers are shown on the right. Controls were performed with lipoic acid omitted (row 2), the acceptor peptide for BirA substituted for LAP (row 3), and the interaction-deficient NRX mutant (row 4). (**B**) ID-PRIME with picolyl azide readout (as in Figure 1C). HEK cells were transfected as in (A), and labeling was performed with 100 µM picolyl azide +500 µM ATP for 15 minutes, followed by detection with copper-catalyzed click chemistry, using 50 µM copper and 20 µM alkyne-AF647. Color schemes and controls are the same as for (A). All scale bars, 10 µm.

Three tandem LAP tags (3xLAP) were introduced onto the *N*-terminus of NLG1, while the 38 kD LplA mutant with improved activity in the secretory pathway (mutations: W37A, T57I, F147L, H267R [29], referred to below as *LplA) was fused to the *N*-terminus of NRX3β after amino acid 36. Lipoic acid ID-PRIME was successfully performed in HEK cultures, with antibody signal detected between transfected cells (Figure 4A). Negative controls with lipoic acid omitted or the LAP tag replaced by AP produced no signal. Like BLINC, ID-PRIME labeling was interaction-dependent because a NRX3β D137A mutation in the *LplA_36_-NRX3β construct eliminated labeling (Figure 4A, bottom row).

Picolyl azide ID-PRIME using these same fusion constructs was also successfully performed in HEK cultures (Figure 4B). Again, negative controls with azide omitted, LAP replaced by AP, or a D137A mutation in NRX3β showed no signal. Here, the ID-PRIME signal (from Alexa Fluor 647-alkyne) was more clearly concentrated at junctions between LplA- and LAP-expressing cells. This is probably because the small molecule detection reagents for picolyl azide ID-PRIME could better access the crowded adhesion junctions compared to antibody detection reagents used for lipoic acid ID-PRIME.

We also compared 1xLAP-NLG1 to 3xLAP-NLG1 for lipoic acid ID-PRIME and found that the tandem LAPs did not boost signal as strikingly as tandem APs did (data not shown), possibly because the 3xLAP tag reduced NLG1 expression at the surface (compared to 1xLAP), or because tandem lipoic acid molecules in close proximity could not be simultaneously accessed by antibodies.

### Lipoic Acid ID-PRIME in Neurons

**Figure 5:**
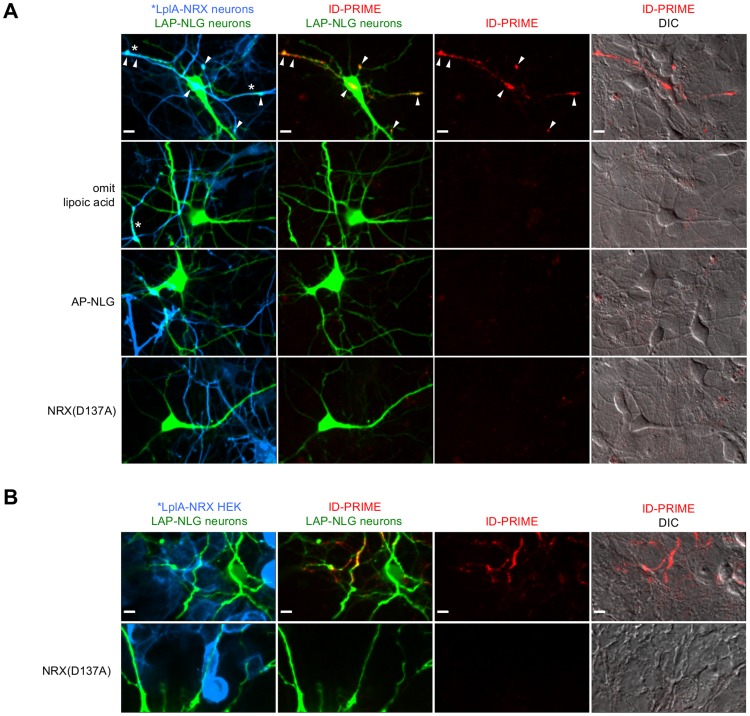
ID-PRIME for imaging neurexin-neuroligin interactions in neuron cultures and in HEK-neuron mixed cultures. (**A**) Lipoic acid ID-PRIME labeling of pure neuron cultures. Dissociated rat hippocampal neurons were separately nucleofected at DIV0 with either 1xLAP-NLG1 plus a Venus transfection marker (shown in green), or *LplA_36_-NRX3β plus a membrane-localized tdTomato transfection marker (shown in blue). The two pools of neurons were mixed and plated. At DIV5, neurons were labeled with lipoic acid and anti-lipoic acid antibody as in Figure 4A. ID-PRIME signal was detected in 22 out of 23 fields of view, and was localized to contact sites (arrow heads, row 1). Negative controls with lipoic acid omitted (row 2), AP-NLG1 in place of LAP-NLG (row 3), or with an interaction deficient mutant of NRX (row 4) are also shown. Asterisks in row 1 and 2 indicate sites where the over-expression of ID-PRIME constructs caused neuronal processes to “zip up”. (**B**) Lipoic acid ID-PRIME labeling of mixed HEK-neuron cultures. HEK cells expressing *LplA_36_-NRX3β and a membrane-localized tdTomato marker (shown in blue) were plated on top of neurons, transfected with lipofectamine at DIV 7 with 3xLAP-NLG1 plus a Venus marker (shown in green). Labeling was performed as in Figure 4A, at DIV8. ID-PRIME signal was detected in 9 out of 11 fields of view in which Venus-expressing neurons contacted Tomato-expressing HEK cells. The bottom row shows a control with a D137A mutation in NRX; no ID-PRIME signal was observed in any field of view. All scale bars, 10 µm.

We proceeded to test labeling in neuron cultures. Using the same nucleofection protocol developed for BLINC, lipoic acid ID-PRIME signal was detected at overlap sites between neurons expressing *LplA_36_-NRX3β and neurons expressing 1xLAP-NLG1 (Figure 5A). Controls with lipoic acid omitted, LAP replaced by AP, or a D137A mutation in NRX eliminated the signal. In contrast to BLINC, ID-PRIME signal was localized to overlapping sites and did not appear to spread outward on the LAP-expressing neuron. We believe this is because the LplA-NRX:LAP-NLG stoichiometry was better matched than the BirA-NRX:AP-NLG stoichiometry in these neuron experiments. This is supported by the observation that *LplA_36_-NRX3β surface expression in neurons after nucleofection was much higher than surface expression of our best BLINC construct, BirA_272_-NRX3β, under identical conditions (Figure S7). This higher expression also helps to explain why lipoic acid ID-PRIME labeling was much more robust and reproducible than BLINC labeling in neurons.

We quantified the sensitivity of lipoic acid ID-PRIME in neurons and found that 38–54% of contact sites between transfected neurons (defined by the intersection of fluorescent protein transfection markers for *LplA_36_-NRX3β and 1xLAP-NLG) contained ID-PRIME signal. The lack of signal at ∼46% of contacts could be due to sensitivity limits of the methodology, or an absence of true NRX-NLG interactions at fluorescent marker intersection sites. Like in our neuron BLINC experiments, ID-PRIME constructs also induced “zipping” of neuronal processes (Figure 5A, marked sites in rows 1 and 2) when overexpressed.

Lipoic acid ID-PRIME also detected the trans-cellular interaction between 3xLAP-NLG1 expressed in neurons and *LplA_36_-NRX3β expressed in overlaid HEK cells (Figure 5B). A D137A mutation in NRX eliminated ID-PRIME signal in this mixed culture configuration.

To introduce reporter plasmids into separate pools of neurons by nucleofection is labor intensive and consumes large numbers of neurons. Lipofection of plated neurons [33] is much simpler and uses fewer cells, so we also attempted lipoic acid ID-PRIME using this strategy. Since lipofection usually transfects a somewhat random and small subset of neurons, and the lipofecting medium can be removed within hours of addition, we surmised that it would be possible, by sequential lipofection, to generate a culture in which some neurons express NRX only, some neurons express NLG only, and some express both. Figure S8 shows neuron cultures lipofected with *LplA_36_-NRX3β at DIV5, and again with LAP-NLG1 one day later, at DIV6. After labeling and antibody staining at DIV7, we detected *trans* signal in many fields of view (evident from ID-PRIME signal on top of overlapping green and blue processes), but contaminating *cis* signal from neurons co-expressing both fusion constructs was also observed in some fields of view. The *cis* signal was generally much stronger than the *trans* signal, and spread over the entire surface of the transfected neuron, instead of being localized to green-blue overlap sites. We conclude that this experimental configuration is useful and much easier to implement, but one must be cautious about interpreting signal as being *trans* or *cis* in origin.

### Picolyl Azide ID-PRIME in Neurons

We also used sequential lipofection of plated hippocampal neurons to test picolyl azide ID-PRIME. Figure S9A shows fluorescent signal from the Alexa Fluor 647-alkyne (AF647-alkyne) used for picolyl azide detection at contact sites between blue LplA-NRX-expressing neurons and green LAP-NLG-expressing neurons. A negative control with picolyl azide omitted showed no labeling. The *trans* picolyl azide ID-PRIME signal was also localized to contact sites, but was weak – considerably weaker than lipoic acid ID-PRIME signal with the same reporter constructs, perhaps because the two-tiered antibody detection of lipoic acid offers signal amplification. Accordingly, we were unable to detect picolyl azide ID-PRIME signal in nucleofected neurons which express the reporters at lower levels than lipofected neurons (data not shown).

Since the fusion site of BirA in NRX3β influenced the efficiency of BLINC, we also prepared a stalk-domain fusion of *LplA in NRX3β at the same site as BirA, (*LplA_272_-NRX3β) and tested this construct for picolyl azide ID-PRIME in lipofected neurons. Figure S9B shows that *LplA_36_-NRX3β and *LplA_272_-NRX3β gave comparable ID-PRIME signals, suggesting that ID-PRIME is less sensitive to the fusion geometry of *LplA, possibly because both fusion constructs were expressed more abundantly than any BirA-NRX3β construct in neurons.

## Discussion

In summary, our work presents three findings: (1) The BLINC methodology introduced in previous work [5] (paper now retracted) could be reproduced in HEK cells, but not in neuron cultures using the constructs and protocols previously described. (2) By re-designing the NRX and NLG fusion constructs and modifying the labeling protocol, we were able to achieve BLINC labeling in neuron cultures. (3) Due to poor surface expression of BirA fusion constructs, our new BLINC protocol was still not robust in neurons, so we developed ID-PRIME for detection of trans-cellular NRX-NLG interactions. ID-PRIME was much more robust than BLINC in neuron cultures and the signal could be read out by either antibodies or small-molecule reagents.

Regarding the first point, the reasons for the lack of reproducibility of BLINC in neurons that we observed were: (i) the CMV promoter plasmids used previously [5], when introduced at DIV0 by nucleofection, expressed only transiently and could not be detected by DIV5. All previously reported experiments were performed in neurons at DIV5 and DIV16 [5], when BLINC reporters were no longer present. (ii) Even after changing the promoter from CMV to CAG to obtain persistent expression after nucleofection, the 1xAP tag on NLG1 was barely detectable above background. (iii) BirA inserted near the *N*-terminus of the mature NRX, as in the previous study [5], did not give detectable BLINC signal in neurons, even when the CAG promoter and a 3xAP-NLG1 were used. (iv) Use of biotin-AMP gave high background signal in neuron BLINC experiments. This reagent was used in all experiments in the previous study [5].

Here, we achieved BLINC labeling in neuron cultures by driving persistent expression with CAG promoters, installing 3xAP in place of 1xAP on NLG, moving BirA to the stalk domain of NRX, and using biotin+ATP instead of biotin-AMP. Nevertheless, we found that BLINC in neurons was not robust and sometimes failed, likely due to poor surface targeting of even our best BirA-NRX fusion. It is, however, a strength of the labeling system that such low, virtually non-detectable levels of surface BirA expression could produce detectable BLINC signal, attesting to the high sensitivity of streptavidin-fluorophore detection. Engineering of the BirA sequence or exploration of alternative fusion sites may improve surface BirA expression in future reporter designs.

In contrast to neurons, BLINC in non-neuronal cells (e.g., HEK) and in mixed neuron-HEK cultures, which comparatively exhibit higher surface expression of BirA constructs, was very reliable and specific. The signal was also interaction-dependent, and well-localized to cell-cell contact sites, and should therefore be a useful method with which to detect and image other trans-cellular protein-protein interactions.

Lastly, we introduce in this study a new methodology for imaging trans-cellular protein complexes using interaction-dependent PRIME (ID-PRIME). In ID-PRIME, an LplA mutant replaces BirA, and LAP replaces AP. The advantages of ID-PRIME over BLINC are two-fold: (1) The *LplA-NRX fusion has much better surface targeting than BirA-NRX (despite its slightly larger size of 38 kD), leading to robust and reproducible signal in neuron cultures that is localized to NRX-NLG contact sites. (2) Using the picolyl azide detection strategy [7] (Figure 1C), NRX-NLG interactions can be read out with small, bright fluorophores that are less likely to introduce trafficking artifacts or be sterically excluded from crowded synaptic regions compared to the streptavidin reagent used for BLINC. This work demonstrated ID-PRIME in three experimental configurations: HEK cultures (Figure 4), mixed HEK-neuron cultures (Figure 5B), and neuron cultures (Figure 5A). Given its advantages over BLINC, ID-PRIME expands the toolkit available for imaging trans-cellular protein-protein interactions in living cells.

There are still some issues to be resolved, however, before ID-PRIME can be a minimally-invasive and faithful tool with which to study physiologically relevant NRX-NLG interactions in neurons. The major concern is that its sensitivity must be improved to the point that signal can be easily detected even when reporter constructs are not overexpressed. This is particularly true for picolyl azide ID-PRIME, which currently has lower sensitivity than lipoic acid ID-PRIME in neurons even though its reagents are more suitable for reporting on synaptic protein complexes. A second concern is that we did not examine the effects of neurexin and neuroligin shedding on BLINC or ID-PRIME signal in this work. Recent studies have shown that the ectodomains of both NRX [34], [35] and NLG [36], [37] may be cleaved by membrane-anchored metalloproteases in a potentially activity-dependent manner. This could complicate the interpretation of BLINC and ID-PRIME data if some fusion constructs of NRX and NLG are more prone to cleavage than wild-type (giving false negatives), or if labeling signal on the cleaved NLG ectodomain that ought to have escaped into the medium is trapped by full-length NRX (giving false positives). We plan to study and address these limitations in future work.

## Supporting Information

Figure S1 Expression of CMV-AP-NLG1 from reference [5] cannot be detected in neurons, but CAG promoter constructs can be detected. The indicated plasmids were introduced by nucleofection, along with a Venus marker (shown in green), into DIV0 dissociated rat hippocampal neurons. At either DIV5 (left), or DIV12 (right), surface AP fusion proteins were labeled with 1 uM exogenous BirA (+ biotin and ATP), followed by streptavidin-AF647 (shown in red), then imaged live. Streptavidin channel intensities are normalized within the DIV5 dataset and within the DIV12 dataset, but not across datasets. Labeling of CMV-1xAP-NLG1 was not detected across 10 transfected cells at DIV5, and 23 transfected cells at DIV12. Labeling of CAG-1xAP-NLG1 was detectable but weak in 4 of 10 transfected cells at DIV5, and 12 of 23 transfected cells at DIV12. Labeling of CAG-3xAP-NLG1 (with three AP tags in tandem) was generally stronger, and detected in 8 of 9 neurons at DIV5, and 12 of 16 transfected neurons at DIV12. On the right, the arrowhead points to a lightly streptavidin-labeled cell that expressed the Venus marker weakly. Scale bars, 10 µm.
(TIF)

Figure S2 Expression of CMV-BirA_64_-NRX1β from reference [5] cannot be detected in neurons after nucleofection. The CMV-BirA_64_-NRX1β plasmid was introduced by nucleofection, along with a Venus marker (shown in green), into DIV0 dissociated rat hippocampal neurons. Anti-*c*-Myc staining was performed on living cells to detect surface expression of the BirA-NRX at DIV5 (top) and DIV12 (bottom). As a positive control, staining was performed in parallel on neurons transfected with the same plasmid, using lipofectamine instead of nucleofection, 1 day before the labeling experiment. In general, we find that lipofection of a plasmid gives much higher expression in neurons than nucleofection of the same plasmid. AF647 channel intensities are normalized within DIV5 and DIV12 datasets, but not across datasets. For samples nucleofected with CMV-BirA_64_-NRX1β following the protocol in reference [5], *c*-Myc staining could not be detected across 13 transfected cells at DIV5 and 16 transfected cells at DIV12. For lipofected control samples, *c*-Myc staining could be detected in 4 of 5 cells at DIV5, and 4 of 7 cells at DIV12. In general, *c*-Myc staining on lipofected neurons was weaker at DIV12 than at DIV5 in this experiment. Scale bars, 10 µm.
(TIF)

Figure S3 The CAG promoter gives more persistent expression of BirA_64_-NRX1β in neurons than the CMV promoter. BirA_64_-NRX1β with either a CMV or CAG promoter was introduced to plated hippocampal neurons at DIV5 using lipofectamine, along with a synaptophysin-YFP marker (shown in green). Expression was detected 4 days later, at DIV9, by anti-*c*-Myc staining on either living neurons (top) or fixed and permeabilized neurons (bottom). *c*-Myc staining background was very high for fixed neurons. Whereas CMV-BirA_64_-NRX1β expression could not be detected across multiple fields of view, CAG-BirA_64_-NRX1β expression was detectable 4 days after lipofection. Note that in Figure S2, lipofected CMV-BirA_64_-NRX1β was detected 1 day rather than 4 days after lipofection. Scale bars, 10 µm.
(TIF)

Figure S4 Trafficking of BirA fusion constructs in HEK and neurons. **(A)** Domain structures of BirA and AP fusions to NRX3β, NRX1β, and NLG1 used in this figure. Construct numbering according to Table S1 is given at right. TM is the transmembrane domain. **(B)** BirA and AP insertion sites in NRX and NLG. A side-on view into the synaptic cleft is shown for the dimeric extracellular domain of NLG1 (amino acids 52-634) in complex with two extracellular domains of NRX1β (amino acids 82-288; colored orange). From PDB 3VKF [38]. Ca^2+^ ions are shown in green. Note that amino acid 288 of NRX1β corresponds to amino acid 259 of NRX3β. **(C)** Trafficking in HEK cells. Cells were transfected with the indicated constructs, fixed and permeabilized, then stained with the indicated antibodies. Fluorescence images are not normalized. Bottom row shows overlay onto DIC images. The BirA tag reduces surface trafficking of NRX1β, NRX3β, and NLG1. **(D)** Trafficking in neurons. Hippocampal neurons were lipofected at DIV11 with the indicated constructs and a Venus co-transfection marker (shown in green). One day later, neurons were stained live with anti-HA antibody to visualize surface expression. **(E)** Same as (D) except that neurons were fixed and permeabilized before staining with anti-HA antibody to visualize total protein pools, rather than surface pools only. Trafficking of NRX3β and NLG1 to processes is impaired when either is fused to BirA. Scale bars, 10 µm.
(TIF)

Figure S5 Synaptic localization of optimized BLINC constructs in neurons. Hippocampal neurons were lipofected with the indicated constructs and synaptic markers at DIV11 and imaged live at DIV12. The pre-synaptic marker synaptophysin-YFP was used at left, and the post-synaptic marker Homer-GFP was used at right, both shown in green. Anti-HA staining was performed on living neurons to visualize surface pools of BirA (left) and AP (right) fusion proteins. In the “merge” panel, yellow indicates sites of red-green overlap. All scale bars, 10 µm.
(TIF)

Figure S6 Use of biotin-AMP for BLINC in neuron cultures generates high imaging background. Hippocampal neurons were nucleofected at DIV0 with BirA_272_-NRX3β plus dsRed (shown in blue), or 3xAP-NLG1 plus Venus (shown in green). The two pools were mixed together and allowed to form contacts. At DIV9, cells were labeled with biotin+ATP, or biotin-AMP ester, as indicated for 5 minutes, then stained with monovalent streptavidin-AF647 (shown in red) for another 5 minutes and imaged live. On the right are images of untreated coverslips. From this experiment we conclude that signal intensities are similar for biotin+ATP, and 2.5 uM biotin-AMP. However, the nonspecific background is higher when using 2.5 uM biotin-AMP. In contrast, the background when using biotin+ATP is the same as for untreated coverslips, i.e., undetectable. Note that the problem of high background with biotin-AMP is observed only for neurons, and is not seen when performing BLINC or exogenous BirA labeling on HEK cells (as in Figure 2B). Scale bars, 10 µm.
(TIF)

Figure S7 Comparison of surface trafficking in neurons for BLINC and ID-PRIME ligase fusion constructs. **(A)** Domain structures of LplA, BirA, and LAP fusion constructs used in this figure and Figures S8 & S9. Construct numbering according to Table S1 is given at right. TM is the transmembrane domain. HA tags are colored red and a linker is colored green. **(B)** Comparison of surface trafficking in neurons for BLINC and ID-PRIME ligase fusion constructs. Hippocampal neurons were nucleofected at DIV0 with *LplA_36_-NRX3β or BirA_272_-NRX3β, plus a membrane tdTomato marker (shown in green). At DIV5, surface expression of each construct was detected by live-cell immunostaining with anti-HA antibody, shown in red at two different intensity levels. *LplA_36_-NRX3β surface expression was easily detected in 19 out of 19 transfected neurons, while BirA_272_-NRX3β surface expression was undetectable in 10 out of 10 transfected neurons. Note that in Figure S4D, surface detection of BirA_272_-NRX3β was performed after lipofection, not nucleofection. Scale bars, 10 µm.
(TIF)

Figure S8 Lipoic acid ID-PRIME with lipofected neuron cultures. Same as Figure 5A, except that constructs were introduced by sequential lipofection into plated hippocampal neurons at DIV5 and DIV6, instead of by nucleofection into separate pools of DIV0 neurons (which ensures complete plasmid segregation). For lipofection, *LplA_36_-NRX3β plus a membrane tdTomato marker (shown in blue) were first introduced at DIV5, then the same cultures were lipofected again at DIV6 with 1xLAP-NLG1 plus a Venus marker (shown in green). All constructs had CAG promoters. At DIV7, lipoic acid ID-PRIME labeling was performed as in Figure 4A. Expression of *LplA-NRX and 1xLAP-NLG in the same neuron (indicated by overlap of green and blue markers) resulted in diffuse *cis* ID-PRIME signal (row 1) much stronger than the trans-cellular ID-PRIME signal (row 2) in the same dish. Trans-cellular ID-PRIME signal was always localized to contact sites (arrow heads). Omission of lipoic acid suppressed both *cis* (row 3) and *trans* (row 4) ID-PRIME signal. Scale bars, 10 µm.
(TIF)

Figure S9 Picolyl azide ID-PRIME in lipofected neuron cultures. **(A)** Same as Figure S8, but with picolyl azide rather than lipoic acid readout. Neurons were transfected with two sequential rounds of lipofection at DIV5 and DIV6. As a result, some neurons express *LplA_36_-NRX3β with a membrane td-Tomato marker, some express 1xLAP-NLG1 with a Venus marker, and some express all four plasmids. Picolyl azide labeling was performed live with 100 µM picolyl azide +500 µM ATP for 20 minutes. Neurons were then fixed, and ligated azide was detected with 1 mM CuSO_4_ and 5 µM alkyne-AF647 for 1 hour. **(B)** Geometry-independence of ID-PRIME signal in neurons. Neurons were transfected as in (A) with two sequential rounds of lipofection at DIV7 and DIV8. In the top row, *LplA_36_-NRX3β (*N*-terminal fusion construct) was used, while in the bottom row, *LplA_272_-NRX3β (stalk fusion construct) was used. Labeling with picolyl azide and alkyne-AF647 was performed as in (A). Scale bars, 10 µm.
(TIF)

Table S1 Genetic constructs used in this work.
(TIF)

Methods S1 Supporting materials and methods.
(DOCX)
