# Correction: Imaging Trans-Cellular Neurexin-Neuroligin Interactions by Enzymatic Probe Ligation

**DOI:** 10.1371/annotation/a92a8d4b-6b2f-4beb-9d4f-4e33239987fd

**Published:** 2013-04-17

**Authors:** Daniel S. Liu, Ken H. Loh, Stephanie S. Lam, Katharine A. White, Alice Y. Ting

In 2007, one of the authors (Ting) filed a patent related to methods that employ lipoic acid ligase to label proteins. While our article reports a method based on lipoic acid ligase, the findings of this study did not constitute the basis of the patent application and as a result, the authors did not originally declare this patent.

We apologize for this omission and would like to disclose that we hold the patent 11/983605 related to methods that employ lipoic acid ligase to label proteins. 

