# Supplementary figures and images for: Imaging Trans-Cellular Neurexin-Neuroligin Interactions by Enzymatic Probe Ligation
Source: PLoS One. 2013 Feb 14;8(2):e52823. doi: 10.1371/journal.pone.0052823 (PMC3573046; doi:10.1371/journal.pone.0052823)

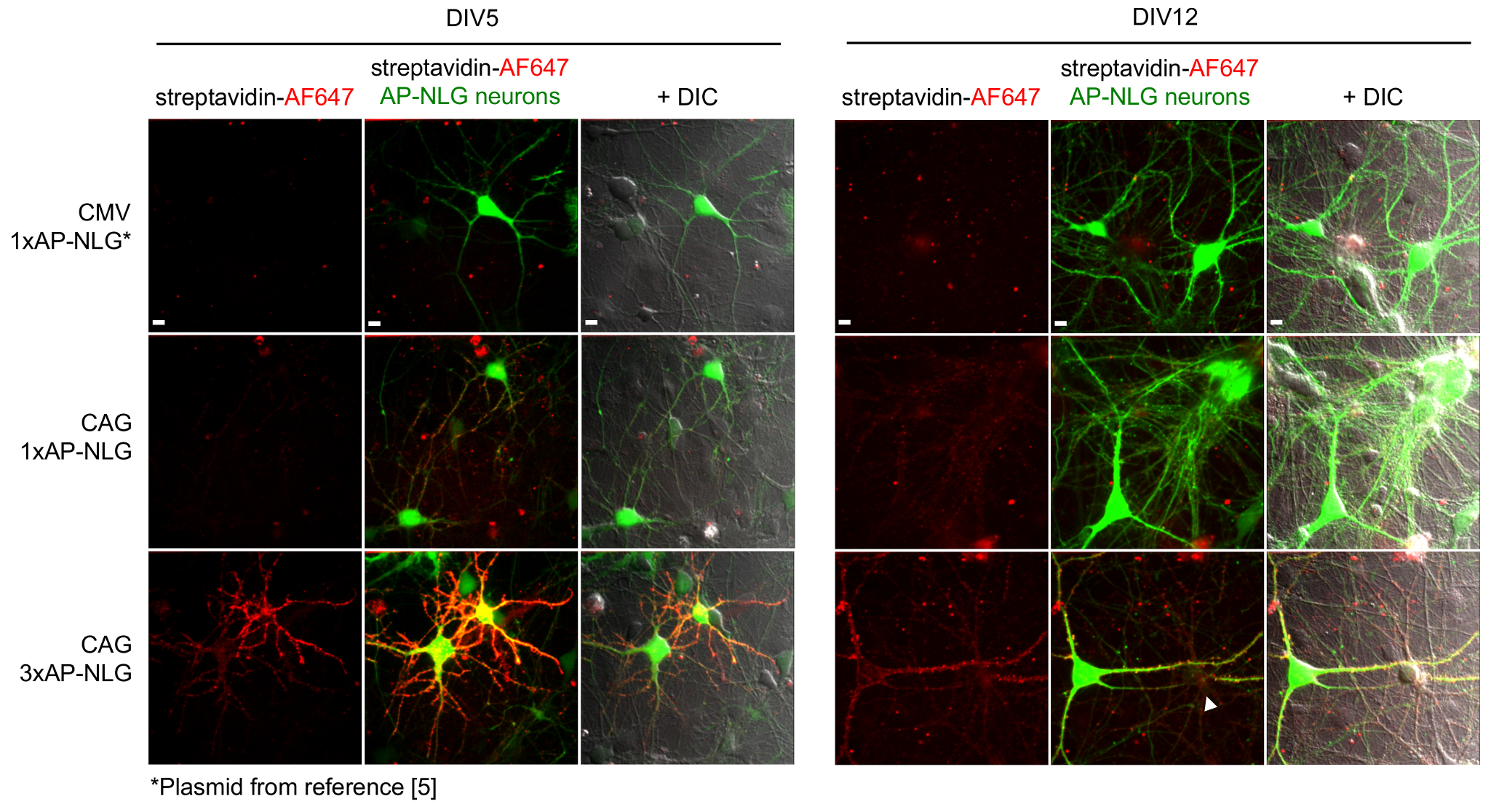

Supplement: Figure S1 — Expression of CMV-AP-NLG1 from reference [5] cannot be detected in neurons, but CAG promoter constructs can be detected. The indicated plasmids were introduced by nucleofection, along with a Venus marker (shown in green), into DIV0 dissociated rat hippocampal neurons. At either DIV5 (left), or DIV12 (right), surface AP fusion proteins were labeled with 1 uM exogenous BirA (+ biotin and ATP), followed by streptavidin-AF647 (shown in red), then imaged live. Streptavidin channel intensities are normalized within the DIV5 dataset and within the DIV12 dataset, but not across datasets. Labeling of CMV-1xAP-NLG1 was not detected across 10 transfected cells at DIV5, and 23 transfected cells at DIV12. Labeling of CAG-1xAP-NLG1 was detectable but weak in 4 of 10 transfected cells at DIV5, and 12 of 23 transfected cells at DIV12. Labeling of CAG-3xAP-NLG1 (with three AP tags in tandem) was generally stronger, and detected in 8 of 9 neurons at DIV5, and 12 of 16 transfected neurons at DIV12. On the right, the arrowhead points to a lightly streptavidin-labeled cell that expressed the Venus marker weakly. Scale bars, 10 µm. (TIF) [file pone.0052823.s001.tif]

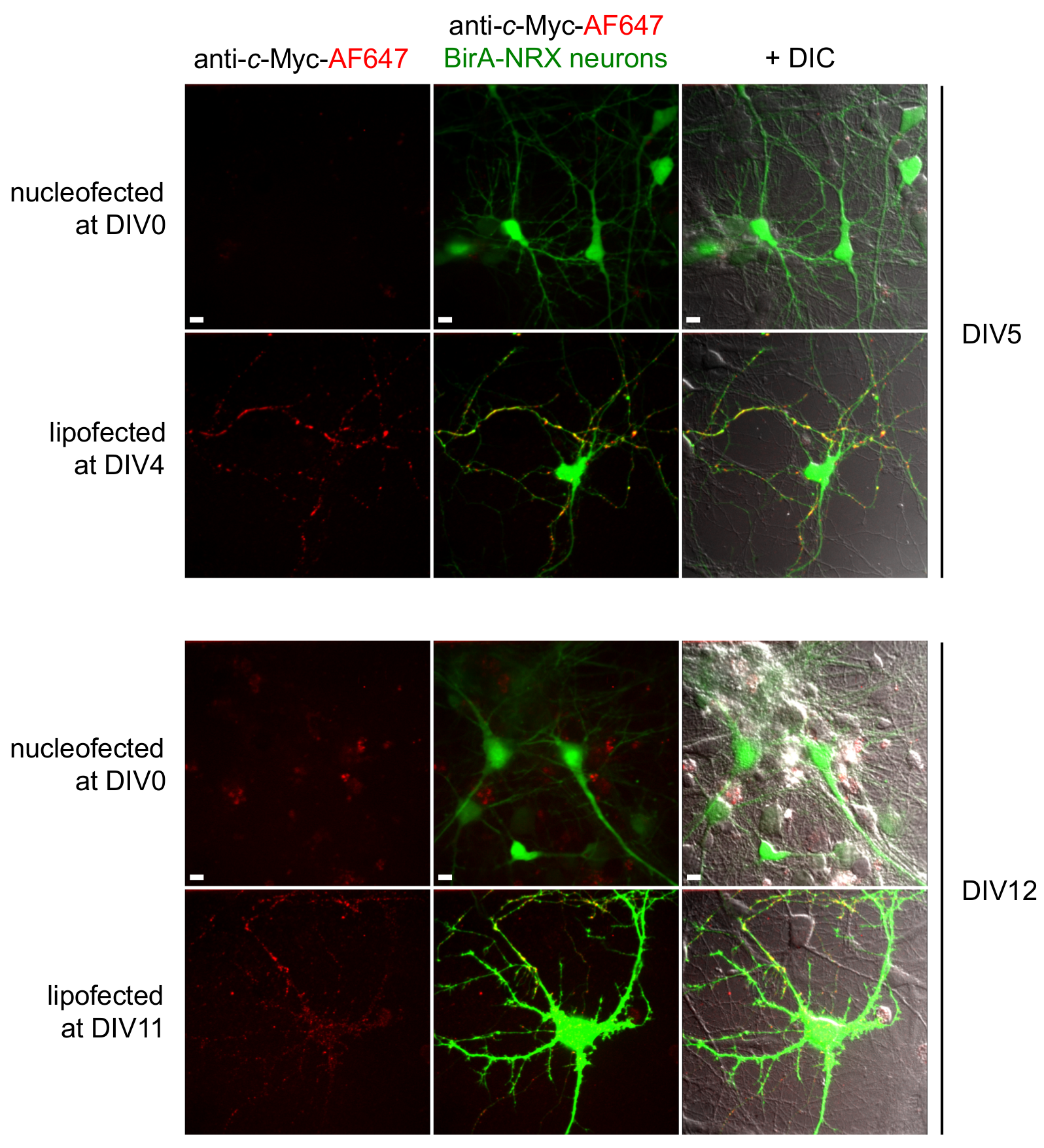

Supplement: Figure S2 — Expression of CMV-BirA64-NRX1β from reference [5] cannot be detected in neurons after nucleofection. The CMV-BirA64-NRX1β plasmid was introduced by nucleofection, along with a Venus marker (shown in green), into DIV0 dissociated rat hippocampal neurons. Anti-c-Myc staining was performed on living cells to detect surface expression of the BirA-NRX at DIV5 (top) and DIV12 (bottom). As a positive control, staining was performed in parallel on neurons transfected with the same plasmid, using lipofectamine instead of nucleofection, 1 day before the labeling experiment. In general, we find that lipofection of a plasmid gives much higher expression in neurons than nucleofection of the same plasmid. AF647 channel intensities are normalized within DIV5 and DIV12 datasets, but not across datasets. For samples nucleofected with CMV-BirA64-NRX1β following the protocol in reference [5], c-Myc staining could not be detected across 13 transfected cells at DIV5 and 16 transfected cells at DIV12. For lipofected control samples, c-Myc staining could be detected in 4 of 5 cells at DIV5, and 4 of 7 cells at DIV12. In general, c-Myc staining on lipofected neurons was weaker at DIV12 than at DIV5 in this experiment. Scale bars, 10 µm. (TIF) [file pone.0052823.s002.tif]

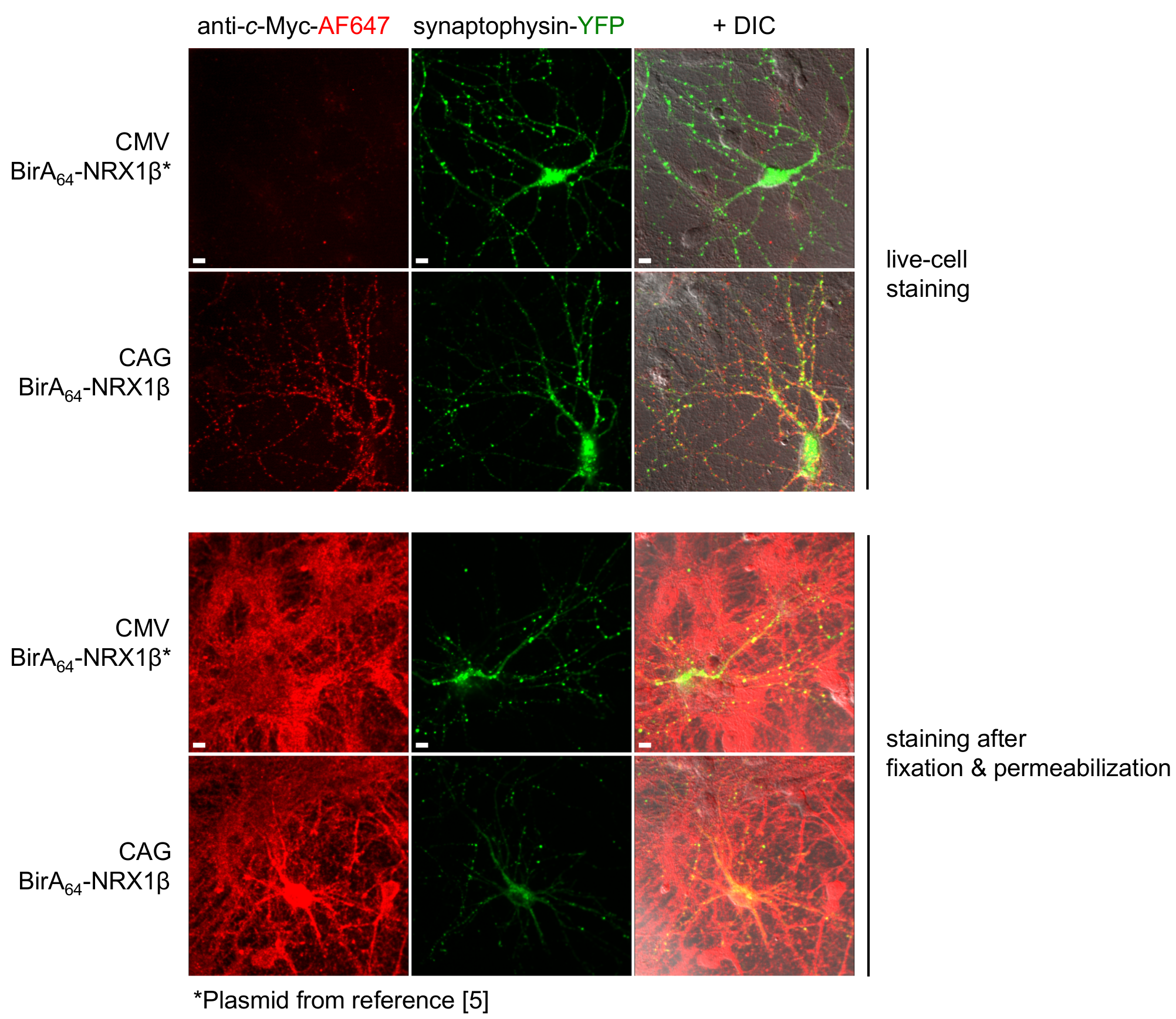

Supplement: Figure S3 — The CAG promoter gives more persistent expression of BirA64-NRX1β in neurons than the CMV promoter. BirA64-NRX1β with either a CMV or CAG promoter was introduced to plated hippocampal neurons at DIV5 using lipofectamine, along with a synaptophysin-YFP marker (shown in green). Expression was detected 4 days later, at DIV9, by anti-c-Myc staining on either living neurons (top) or fixed and permeabilized neurons (bottom). c-Myc staining background was very high for fixed neurons. Whereas CMV-BirA64-NRX1β expression could not be detected across multiple fields of view, CAG-BirA64-NRX1β expression was detectable 4 days after lipofection. Note that in Figure S2, lipofected CMV-BirA64-NRX1β was detected 1 day rather than 4 days after lipofection. Scale bars, 10 µm. (TIF) [file pone.0052823.s003.tif]

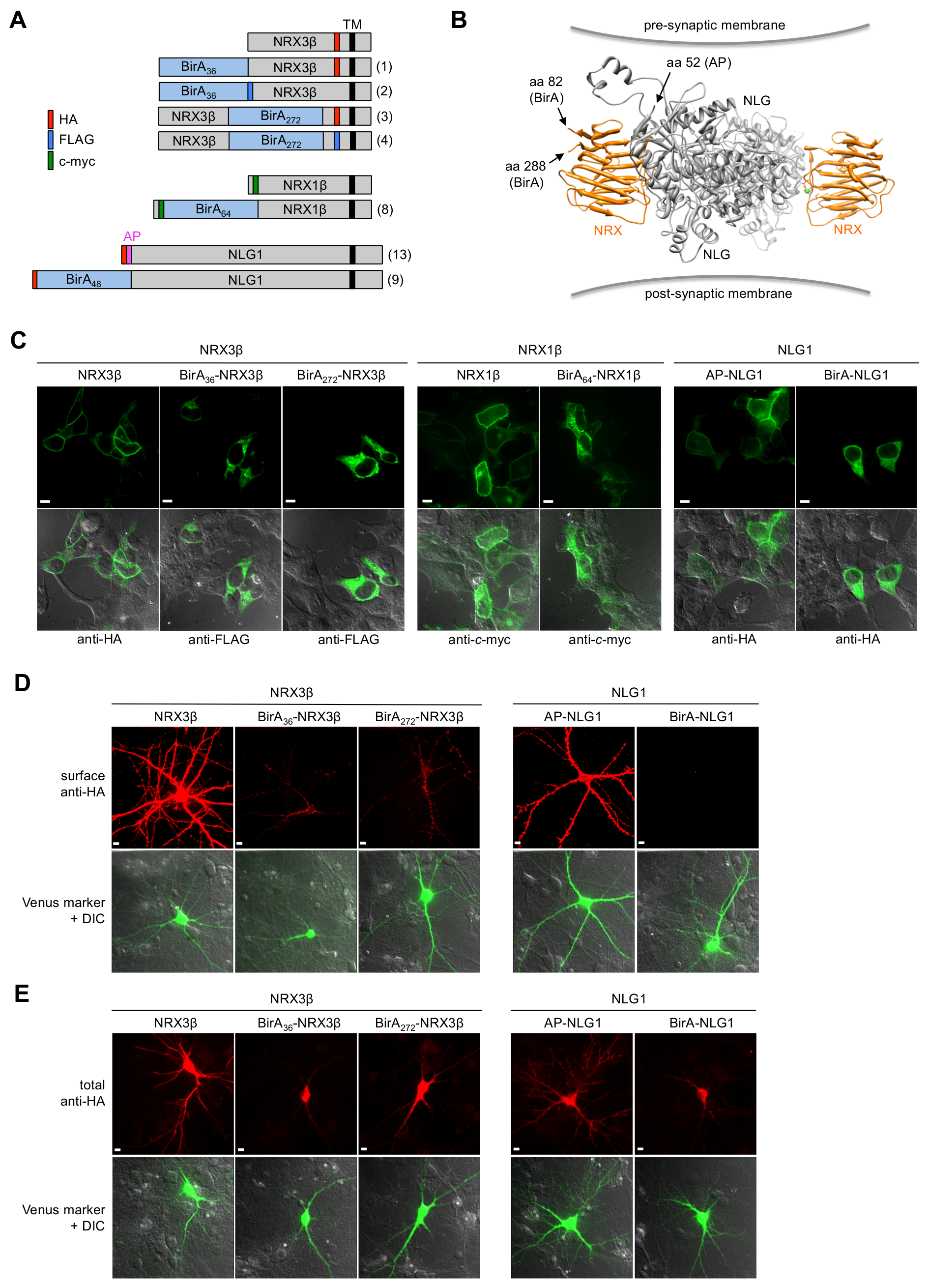

Supplement: Figure S4 — Trafficking of BirA fusion constructs in HEK and neurons. (A) Domain structures of BirA and AP fusions to NRX3β, NRX1β, and NLG1 used in this figure. Construct numbering according to Table S1 is given at right. TM is the transmembrane domain. (B) BirA and AP insertion sites in NRX and NLG. A side-on view into the synaptic cleft is shown for the dimeric extracellular domain of NLG1 (amino acids 52-634) in complex with two extracellular domains of NRX1β (amino acids 82-288; colored orange). From PDB 3VKF [38]. Ca2+ ions are shown in green. Note that amino acid 288 of NRX1β corresponds to amino acid 259 of NRX3β. (C) Trafficking in HEK cells. Cells were transfected with the indicated constructs, fixed and permeabilized, then stained with the indicated antibodies. Fluorescence images are not normalized. Bottom row shows overlay onto DIC images. The BirA tag reduces surface trafficking of NRX1β, NRX3β, and NLG1. (D) Trafficking in neurons. Hippocampal neurons were lipofected at DIV11 with the indicated constructs and a Venus co-transfection marker (shown in green). One day later, neurons were stained live with anti-HA antibody to visualize surface expression. (E) Same as (D) except that neurons were fixed and permeabilized before staining with anti-HA antibody to visualize total protein pools, rather than surface pools only. Trafficking of NRX3β and NLG1 to processes is impaired when either is fused to BirA. Scale bars, 10 µm. (TIF) [file pone.0052823.s004.tif]

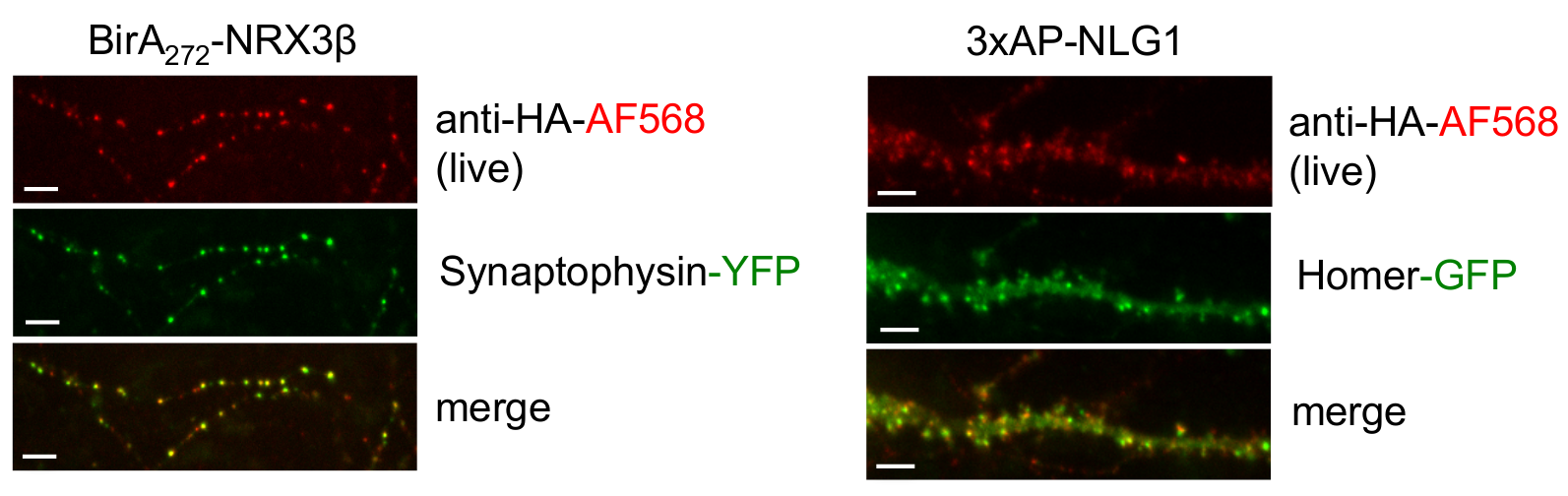

Supplement: Figure S5 — Synaptic localization of optimized BLINC constructs in neurons. Hippocampal neurons were lipofected with the indicated constructs and synaptic markers at DIV11 and imaged live at DIV12. The pre-synaptic marker synaptophysin-YFP was used at left, and the post-synaptic marker Homer-GFP was used at right, both shown in green. Anti-HA staining was performed on living neurons to visualize surface pools of BirA (left) and AP (right) fusion proteins. In the “merge” panel, yellow indicates sites of red-green overlap. All scale bars, 10 µm. (TIF) [file pone.0052823.s005.tif]

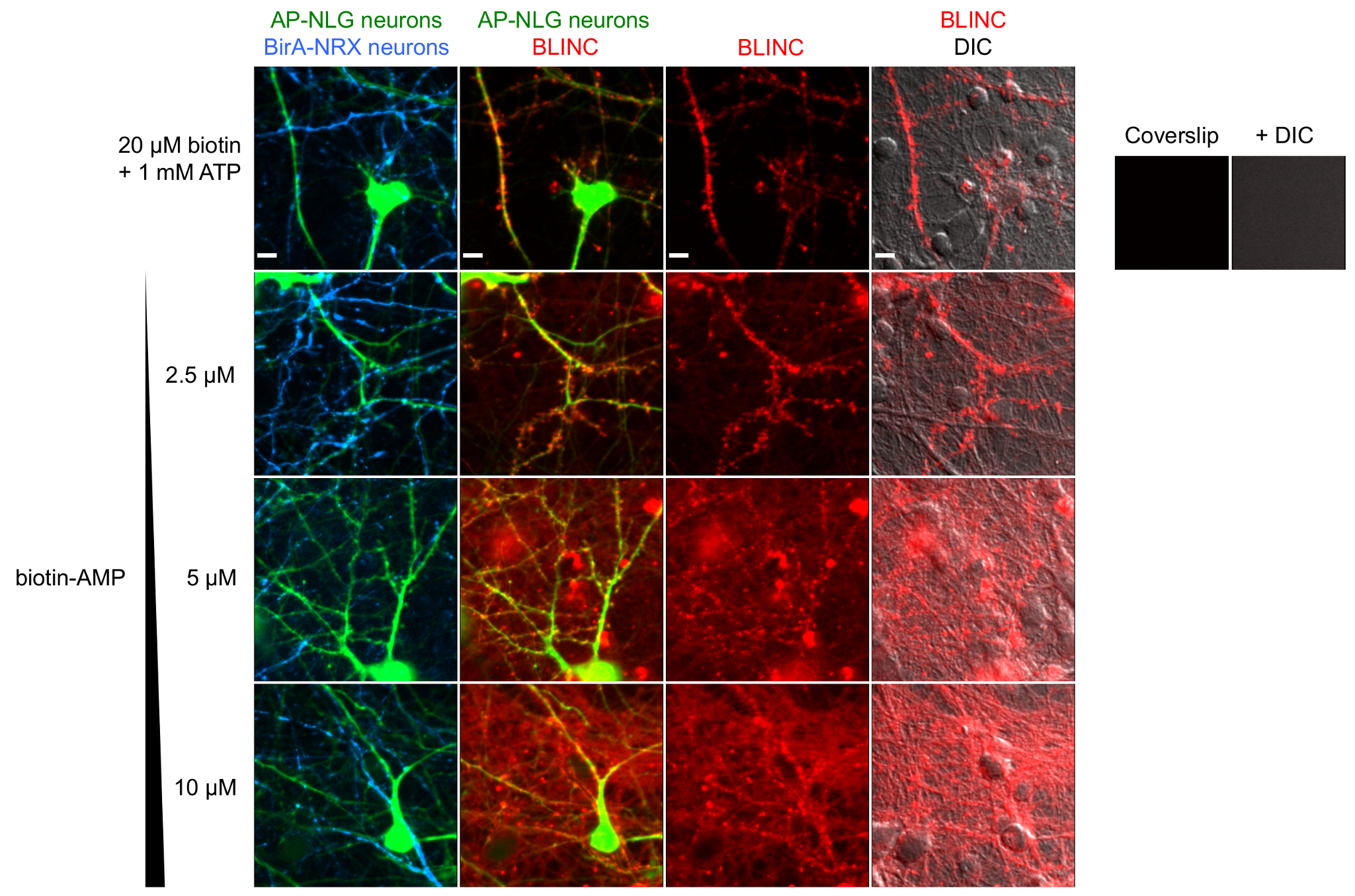

Supplement: Figure S6 — Use of biotin-AMP for BLINC in neuron cultures generates high imaging background. Hippocampal neurons were nucleofected at DIV0 with BirA272-NRX3β plus dsRed (shown in blue), or 3xAP-NLG1 plus Venus (shown in green). The two pools were mixed together and allowed to form contacts. At DIV9, cells were labeled with biotin+ATP, or biotin-AMP ester, as indicated for 5 minutes, then stained with monovalent streptavidin-AF647 (shown in red) for another 5 minutes and imaged live. On the right are images of untreated coverslips. From this experiment we conclude that signal intensities are similar for biotin+ATP, and 2.5 uM biotin-AMP. However, the nonspecific background is higher when using 2.5 uM biotin-AMP. In contrast, the background when using biotin+ATP is the same as for untreated coverslips, i.e., undetectable. Note that the problem of high background with biotin-AMP is observed only for neurons, and is not seen when performing BLINC or exogenous BirA labeling on HEK cells (as in Figure 2B). Scale bars, 10 µm. (TIF) [file pone.0052823.s006.tif]

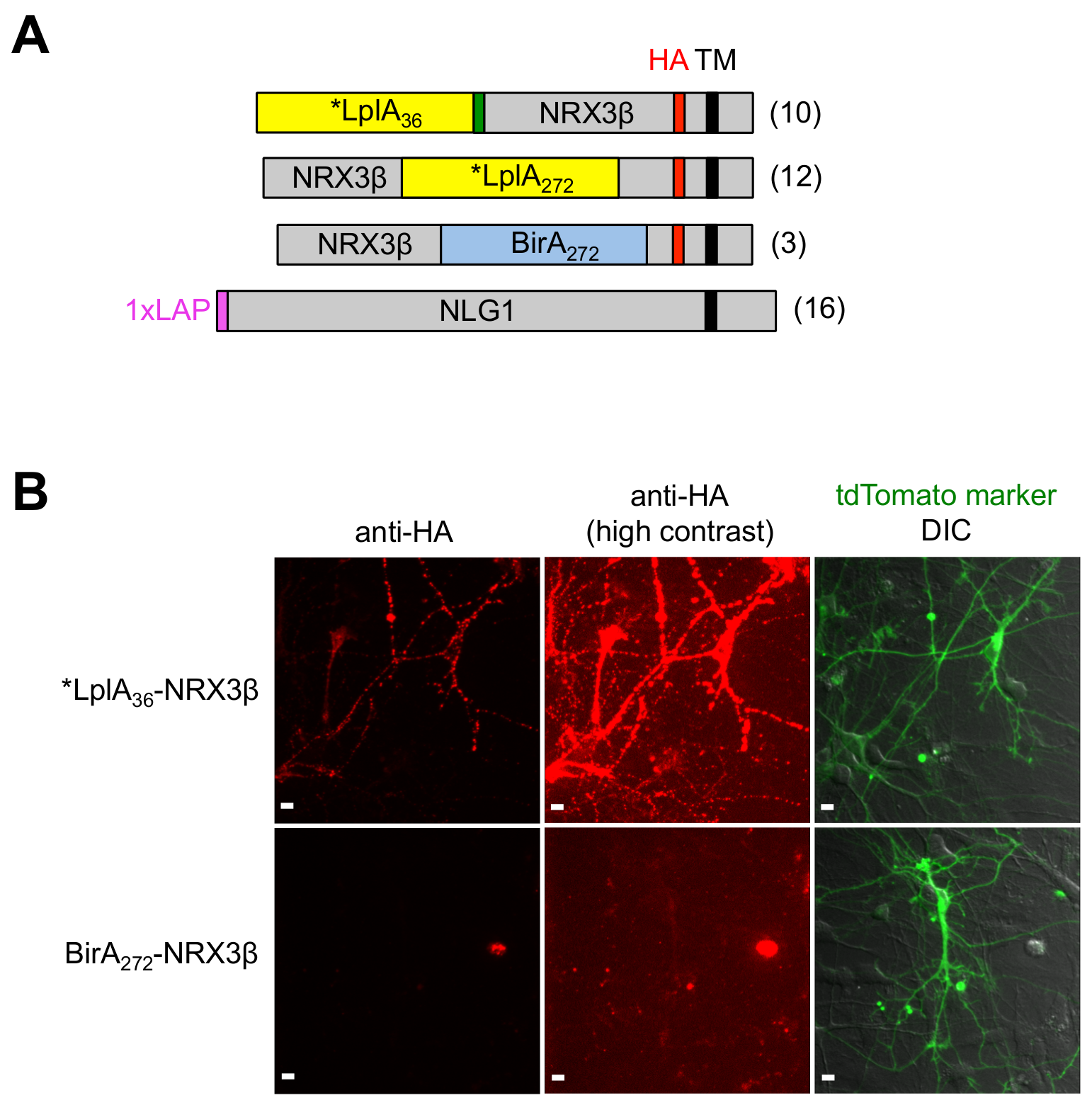

Supplement: Figure S7 — Comparison of surface trafficking in neurons for BLINC and ID-PRIME ligase fusion constructs. (A) Domain structures of LplA, BirA, and LAP fusion constructs used in this figure and Figures S8 & S9. Construct numbering according to Table S1 is given at right. TM is the transmembrane domain. HA tags are colored red and a linker is colored green. (B) Comparison of surface trafficking in neurons for BLINC and ID-PRIME ligase fusion constructs. Hippocampal neurons were nucleofected at DIV0 with *LplA36-NRX3β or BirA272-NRX3β, plus a membrane tdTomato marker (shown in green). At DIV5, surface expression of each construct was detected by live-cell immunostaining with anti-HA antibody, shown in red at two different intensity levels. *LplA36-NRX3β surface expression was easily detected in 19 out of 19 transfected neurons, while BirA272-NRX3β surface expression was undetectable in 10 out of 10 transfected neurons. Note that in Figure S4D, surface detection of BirA272-NRX3β was performed after lipofection, not nucleofection. Scale bars, 10 µm. (TIF) [file pone.0052823.s007.tif]

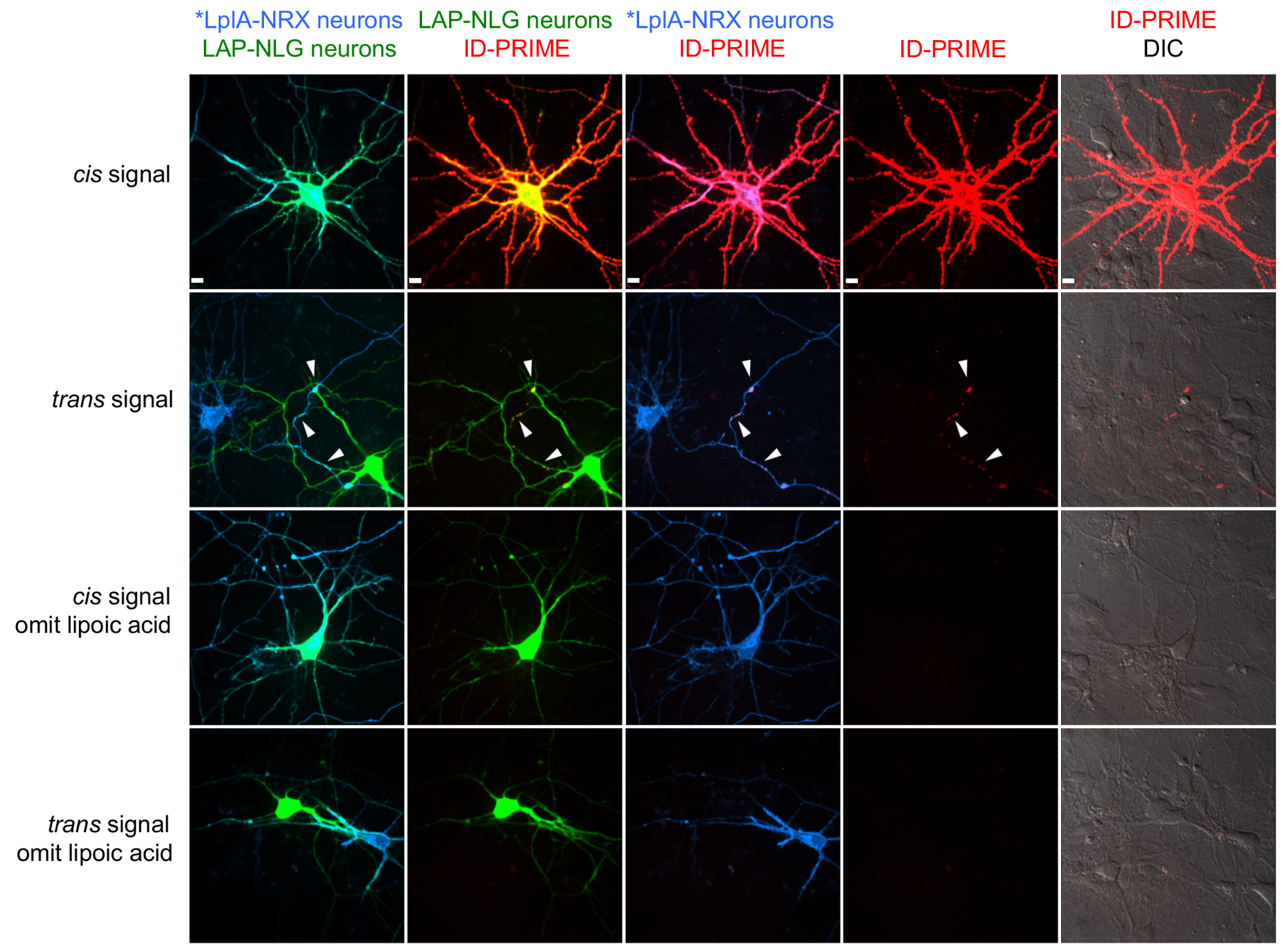

Supplement: Figure S8 — Lipoic acid ID-PRIME with lipofected neuron cultures. Same as Figure 5A, except that constructs were introduced by sequential lipofection into plated hippocampal neurons at DIV5 and DIV6, instead of by nucleofection into separate pools of DIV0 neurons (which ensures complete plasmid segregation). For lipofection, *LplA36-NRX3β plus a membrane tdTomato marker (shown in blue) were first introduced at DIV5, then the same cultures were lipofected again at DIV6 with 1xLAP-NLG1 plus a Venus marker (shown in green). All constructs had CAG promoters. At DIV7, lipoic acid ID-PRIME labeling was performed as in Figure 4A. Expression of *LplA-NRX and 1xLAP-NLG in the same neuron (indicated by overlap of green and blue markers) resulted in diffuse cis ID-PRIME signal (row 1) much stronger than the trans-cellular ID-PRIME signal (row 2) in the same dish. Trans-cellular ID-PRIME signal was always localized to contact sites (arrow heads). Omission of lipoic acid suppressed both cis (row 3) and trans (row 4) ID-PRIME signal. Scale bars, 10 µm. (TIF) [file pone.0052823.s008.tif]

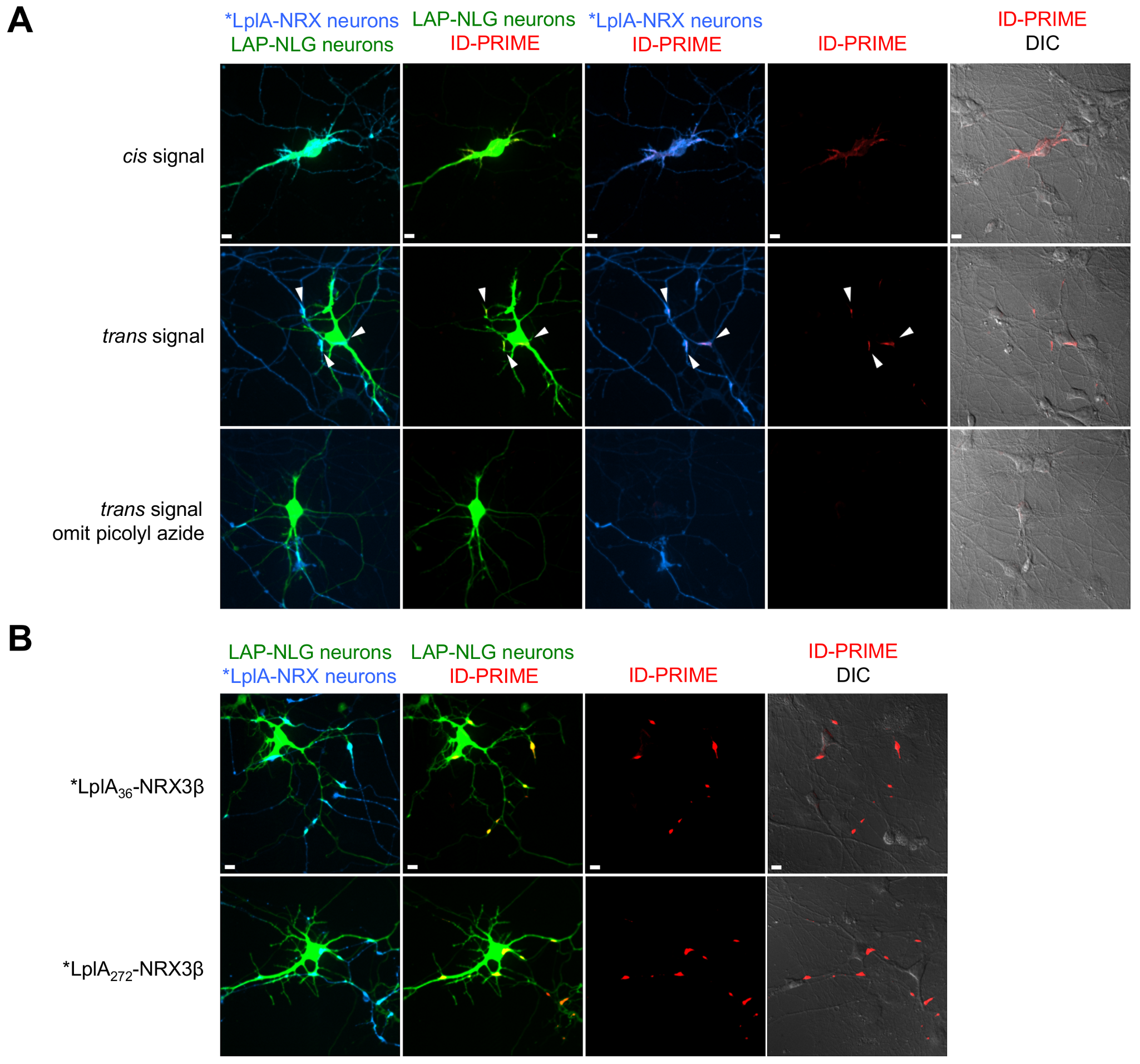

Supplement: Figure S9 — Picolyl azide ID-PRIME in lipofected neuron cultures. (A) Same as Figure S8, but with picolyl azide rather than lipoic acid readout. Neurons were transfected with two sequential rounds of lipofection at DIV5 and DIV6. As a result, some neurons express *LplA36-NRX3β with a membrane td-Tomato marker, some express 1xLAP-NLG1 with a Venus marker, and some express all four plasmids. Picolyl azide labeling was performed live with 100 µM picolyl azide +500 µM ATP for 20 minutes. Neurons were then fixed, and ligated azide was detected with 1 mM CuSO4 and 5 µM alkyne-AF647 for 1 hour. (B) Geometry-independence of ID-PRIME signal in neurons. Neurons were transfected as in (A) with two sequential rounds of lipofection at DIV7 and DIV8. In the top row, *LplA36-NRX3β (N-terminal fusion construct) was used, while in the bottom row, *LplA272-NRX3β (stalk fusion construct) was used. Labeling with picolyl azide and alkyne-AF647 was performed as in (A). Scale bars, 10 µm. (TIF) [file pone.0052823.s009.tif]

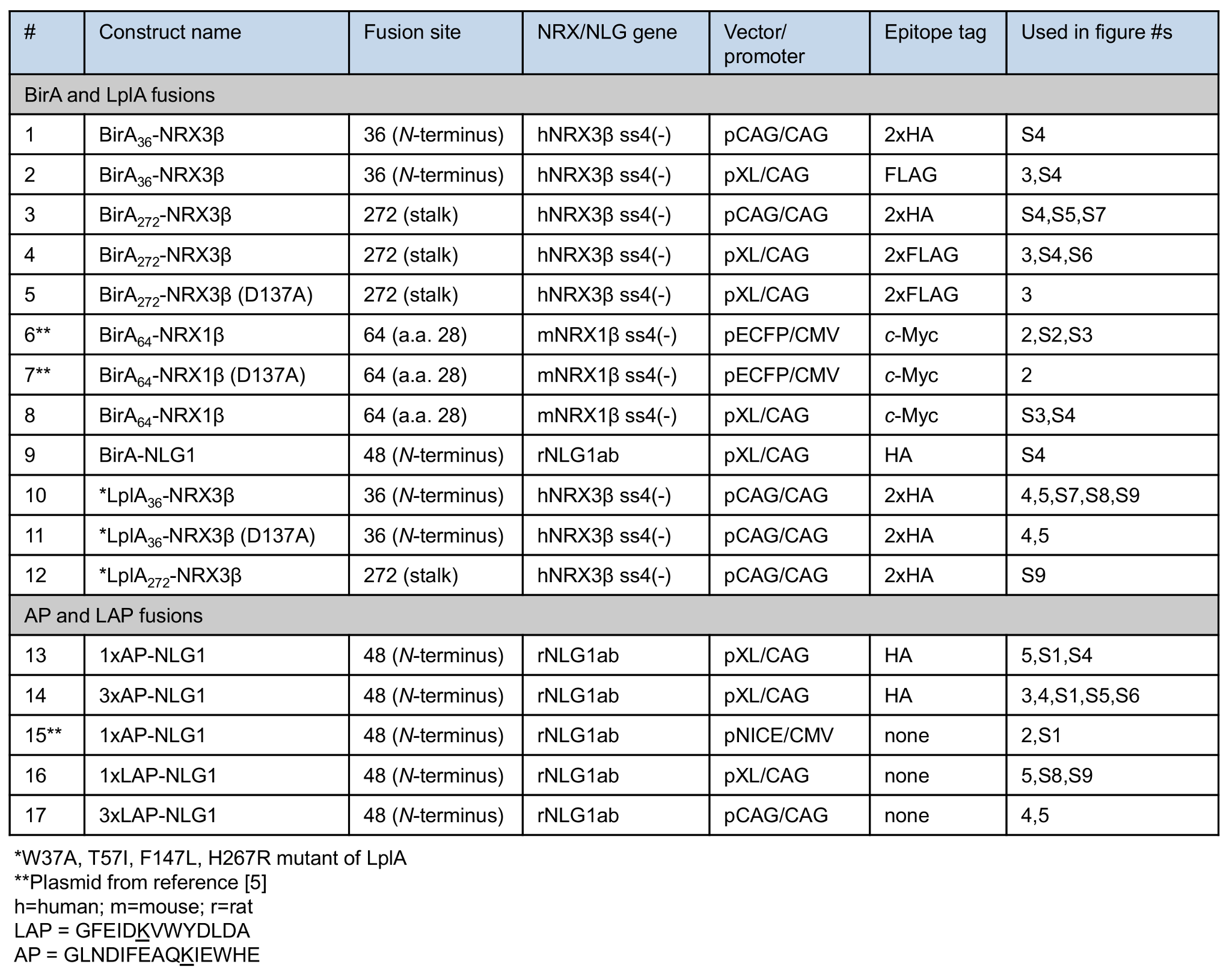

Supplement: Table S1 — Genetic constructs used in this work. (TIF) [file pone.0052823.s010.tif]
