# Supplementary material for: Imaging Trans-Cellular Neurexin-Neuroligin Interactions by Enzymatic Probe Ligation
Source: PLoS One. 2013 Feb 14;8(2):e52823. doi: 10.1371/journal.pone.0052823 (PMC3573046; doi:10.1371/journal.pone.0052823)
Supplement: Methods S1 — Supporting materials and methods. (DOCX) [file pone.0052823.s011.docx]

**Supporting Materials and Methods**

**Imaging Trans-Cellular Neurexin-Neuroligin Interactions by Enzymatic Probe Ligation**

Daniel S. Liu^1^, Ken H. Loh^1^, Stephanie S. Lam, and Alice Y. Ting^*^

Department of Chemistry, Massachusetts Institute of Technology, Cambridge, MA 02139, USA

^1^These authors contributed equally to this work.

^*^Correspondence to: ating@mit.edu

Synthesis of biotin-AMP

122.0 mg biotin (0.50 mmol, gift from Tanabe USA) and 200.0 mg adenosine monophosphate disodium (0.51 mmol) were partially dissolved in a mixture of 2 mL H_2_O, 1 mL *N,N’*-dimethylformamide, and 200 μL pyridine at 4 °C. 300 mg 1-ethyl-3-(3-dimethylaminopropyl)carbodiimide hydrochloride (EDC) was added and the reaction was stirred at 4 °C for 2 hours. After approximately 10 min. of stirring the cloudy mixture clarified into a colorless solution. After 2 hours, the product was precipitated with 40 volumes of room temperature isopropanol. Precipitated solids were isolated by centrifugation in 50 mL conical tubes at 8000 g and washed with 1 volume of isopropanol. After the supernatant was decanted, the remaining solids were dried under vacuum to give a white powder. Approximately 50 mg of the powder was dissolved in H_2_O and purified by reverse phase HPLC on a C_18_ column on a 10-90% acetonitrile linear gradient over 20 min. The product eluted at 11 min. ESI-MS (-) found 572.3.

Wild-type streptavidin, Alexa Fluor 647 conjugate

Wild-type streptavidin containing one His_6_ tag on each subunit was prepared as previously described and stored in phosphate buffered saline, pH 7.4 [[1](#_ENREF_1)]. 300 uL protein at 1.1 mg/mL was basified with 30 uL 1 M sodium bicarbonate and reacted with 10 eq. of Alexa Fluor 647 succinimidyl ester (Life Technologies) from a 10 mg/mL DMSO stock solution at room temperature for 60 min. with continuous agitation. The streptavidin-dye conjugate was purified by size exclusion chromatography on a NAP-10 column (GE Healthcare) developed in Dulbecco’s phosphate buffered saline, pH 7.4.

Monovalent streptavidin, Alexa Fluor 568 and Alexa Fluor 647 conjugates

Monovalent streptavidin was prepared as previously described and stored in phosphate buffered saline, pH 7.4 [[1](#_ENREF_1)]. 300 μL protein at 1.7 mg/mL was basified with 30 μL 1 M sodium bicarbonate and reacted with 10 eq. of either Alexa Fluor 568 succinimidyl ester or Alexa Fluor 647 succinimidyl ester (Life Technologies) at room temperature for 90 min. then purified as described above for the wild-type streptavidin Alexa Fluor 647 conjugate.

(Specific plasmid constructs as listed in Table S1 are referenced in bold brackets.)

BLINC in mixed HEK-neuron cultures (Figure 3B)

One day prior to co-plating of HEK and neurons, HEK cells were lipofected with either BirA_272_-NRX3β **(4)** or BirA_272_-NRX3β D137A **(5)** and a dsRed marker. The next day, DIV10 hippocampal neurons were lipofected with 3xAP-NLG1 **(14)** and a Venus marker. Approximately 10 hours later, neurons were incubated with 0.1 U/mL of sterile DNaseI (New England Biolabs) for one hour. HEK cells were trypsinized and plated on top of lipofected neurons. BLINC labeling was carried out 24 hours after seeding HEK and allowing contacts to form, using the same protocol as for pure HEK and pure neuron cultures.

Lipoic acid ID-PRIME in mixed HEK-neuron cultures (Figure 5B)

A similar protocol to Figure 5A was used, except HEK cells were lipofected with either *LplA_36_-NRX3β **(10)** or *LplA_36_-NRX3β D137A **(11)** and a tdTomato-membrane marker. Neurons were lipofected with 3xLAP-NLG1 **(17)** and a Venus marker. ID-PRIME labeling using lipoic acid was carried out 24 hours after seeding HEK and allowing contacts to form, using the same protocol as for pure HEK and pure neuron cultures.

Detection of AP-NLG in neurons after nucleofection (Figure S1)

Hippocampal neurons were nucleofected at DIV0 with a Venus marker and either CMV-1xAP-NLG1 **(15)**, or CAG-1xAP-NLG1 **(13)**, or CAG-3xAP-NLG1 **(14)**. At DIV5 or DIV12, neurons were labeled with 20 μM biotin, 1 mM ATP, 1.25 mM magnesium acetate and 1 μM BirA in growth medium for 15 min. at 37 °C. Cells were then rinsed three times in Tyrode’s buffer (145 mM NaCl, 1.25 mM CaCl_2_, 3 mM KCl, 1.25 mM MgCl_2_, 0.5 mM NaH_2_PO_4_, 10 mM glucose, 10 mM HEPES, pH 7.4) and subsequently stained with a streptavidin-AF647 conjugate in Tyrode’s buffer for 5 min. at 37 °C. Cells were imaged live after two further rinses in the same buffer.

Comparing CAG and CMV promoters for BirA-NRX expression in neurons (Figure S3)

Hippocampal neurons were lipofected at DIV5 with a synaptophysin-YFP marker and either CMV-BirA_64_-NRX1β **(6)** or CAG-BirA_64_-NRX1β **(8)**. At DIV9, cells were stained with anti-*c*-Myc antibody as described in Materials and Methods and imaged live. Alternatively, cells were fixed with 4% (v/v) formaldehyde in Tyrode’s buffer for 30 min. at room temperature, then blocked with Dulbecco’s phosphate buffered saline (Gibco) containing 0.5% (w/v) casein for 1 hour at room temperature. Immunofluorescence staining was performed with anti-*c*-Myc antibody, in the same way as for live cell cultures, except that it was carried out at room temperature.

Trafficking of BirA fusion constructs in HEK (Figure S4C)

HEK cells were lipofected with the respective NRX/NLG fusion **(2, 4, 8, 13, and 9)**. After 24 hours, cells were fixed with 4% formaldehyde in Tyrode’s buffer, then subsequently permeabilized with cold methanol at -20 °C. Cells were blocked for 1 hour in Tyrode’s buffer supplemented with 0.5% (w/v) casein, followed by primary antibody detection for 1 hour in the same buffer. A 1:200 dilution of one of the following antibodies was used, depending on the epitope tag: mouse anti-*c*-Myc antibody (Calbiochem), rabbit anti-HA antibody (Rockland), or mouse anti-FLAG M2 antibody (Agilent). Cells were then rinsed three times with Tyrode’s buffer and subsequently stained with the corresponding secondary antibodies: goat anti-mouse AF488, goat anti-rabbit AF488 or goat anti-rabbit AF568 (Molecular Probes). Cells were rinsed a further three times with Tyrode’s buffer before imaging.

Trafficking of BirA fusion constructs in neurons (Figures S4D and S4E)

Neurons were lipofected with the respective NRX/NLG fusion **(1, 3, 13, and 9)**.

For live cell immunofluorescence, cells were incubated with a 1:200 dilution of rabbit anti-HA antibody (Rockland) in Tyrode’s buffer supplemented with 0.5% (w/v) casein for 15 min. at 37 °C. Cell were rinsed three times with Tyrode’s buffer, and subsequently stained with the corresponding secondary antibody: goat anti-rabbit AF568 (Molecular probes) in Tyrode’s buffer supplemented with 0.5% (w/v) casein for 5 min. at 37 °C. Cells were rinsed three times with Tyrode’s buffer before imaging live at room temperature.

For fixed cell immunofluorescence, a protocol similar to that used for Figure S4C was used. Antibody and dilution used are the same as those used for live cell immunofluorescence.

Localization of optimized BLINC constructs. (Figure S5)

Hippocampal neurons were lipofected at DIV11 with BirA_272_-NRX3β **(3)** and synaptophysin-YFP (pre-synaptic marker) or 3xAP-NLG1 **(14)** and Homer-GFP (post-synaptic marker). At DIV12, live cell immunofluorescence detection of the HA epitope was carried out using the same protocol as described for Figure S4D.

Background in neuron cultures with biotin-AMP (Figure S6)

Hippocampal neurons were nucleofected at DIV0 with either a Venus marker plus 3xAP-NLG1 **(14)**, or a membrane tdTomato marker plus BirA_272_-NRX3β **(4)**, then mixed and allowed to form contacts. At DIV9 cells were either stained for BLINC with biotin + ATP for 5 min. as described in Materials and Methods (except that 1 mM ATP was used), or with variable concentrations of biotin-AMP for the same amount of time, then detected with monovalent streptavidin-AF647 conjugate and imaged live. Coverslips with no adherent neurons were treated with biotin + ATP in the same way and imaged.

Comparing BirA-NRX and LplA-NRX trafficking to the neuron cell surface (Figure S7B)

Hippocampal neurons were nucleofected at DIV0 with *LplA_36_-NRX3β **(10)** or BirA_272_-NRX3β **(3)**, plus a membrane tdTomato marker. At DIV5, surface expression of the respective constructs was detected by live-cell immunofluorescence staining with a rabbit anti-HA antibody (Rockland), followed by a goat anti-rabbit-AF568 conjugate (Life Technologies), using the same protocol used for Figure S4D.

ID-PRIME with lipofected neuron cultures (Figures S8 and S9A)

Hippocampal neurons were lipofected at DIV5 with *LplA_36_-NRX3β **(10)** plus a membrane tdTomato marker, then the same cultures were lipofected again at DIV6 with 1xLAP-NLG1 **(16)** plus a Venus marker. ID-PRIME labeling using lipoic acid (live cell) and picolyl azide (fixed cell) was carried out as described in Materials and Methods.

Comparison of different LplA insertion sites for picolyl azide ID-PRIME (Figure S9B)

Hippocampal neurons were lipofected at DIV7 with *LplA_36_-NRX3β **(10)** or *LplA_272_-NRX3β **(12)** plus a membrane tdTomato marker, then the same cultures were lipofected again at DIV8 with 1xLAP-NLG1 **(16)** plus a Venus marker. ID-PRIME labeling using picolyl azide (fixed cell) was carried out as described in Materials and Methods. **References**

1. Howarth M, Ting AY (2008) Imaging proteins in live mammalian cells with biotin ligase and monovalent streptavidin. Nat Protoc 3: 534-545.
